# Mitotic Poisons in Research and Medicine

**DOI:** 10.3390/molecules25204632

**Published:** 2020-10-12

**Authors:** Jan Škubník, Michal Jurášek, Tomáš Ruml, Silvie Rimpelová

**Affiliations:** 1 Department of Biochemistry and Microbiology, University of Chemistry and Technology in Prague, Technická 3, 166 28, Prague 6, Czech Republic; jan.skubnik@vscht.cz (J.Š.); tomas.ruml@vscht.cz (T.R.); 2 Department of Chemistry of Natural Compounds, University of Chemistry and Technology in Prague, Technická 3, 166 28, Prague 6, Czech Republic; michal.jurasek@vscht.cz

**Keywords:** mitotic poisons, cancer treatment, clinical trials, colchicine, cytotoxicity, docetaxel, paclitaxel, Taxol

## Abstract

Cancer is one of the greatest challenges of the modern medicine. Although much effort has been made in the development of novel cancer therapeutics, it still remains one of the most common causes of human death in the world, mainly in low and middle-income countries. According to the World Health Organization (WHO), cancer treatment services are not available in more then 70% of low-income countries (90% of high-income countries have them available), and also approximately 70% of cancer deaths are reported in low-income countries. Various approaches on how to combat cancer diseases have since been described, targeting cell division being among them. The so-called mitotic poisons are one of the cornerstones in cancer therapies. The idea that cancer cells usually divide almost uncontrolled and far more rapidly than normal cells have led us to think about such compounds that would take advantage of this difference and target the division of such cells. Many groups of such compounds with different modes of action have been reported so far. In this review article, the main approaches on how to target cancer cell mitosis are described, involving microtubule inhibition, targeting aurora and polo-like kinases and kinesins inhibition. The main representatives of all groups of compounds are discussed and attention has also been paid to the presence and future of the clinical use of these compounds as well as their novel derivatives, reviewing the finished and ongoing clinical trials.

## 1. Introduction

According to the World Health Organization (WHO), in 2018, 9.6 million of patients died of a form of cancer, which represents the cause of approximately every sixth death (70% of cancer deaths reported in low-income countries [1]). Owing to improvement of cancer diagnostics and development of novel anticancer drugs, the cancer mortality rate has been slightly decreasing, recently. The idea of one universal drug capable of treating all types of cancer diseases is not viable anymore. Nowadays, the attention is rather focused on the development of novel, modern methods leading to highly personalized medicine for cancer treatment; next generation sequencing is one good example of such approach enabling one to decipher the cause of a disease and facilitating one to choose a tailor-made treatment [2]. On the most recent list (2019) of essential drugs published by WHO, there is only ca. fifty anticancer drugs, but in reality, there exists even more substances exerting good anticancer properties, many of them still waiting to be discovered [3]. One of the proven general approaches on how to eliminate cancer cells is to target their division. Substances targeting the mitotic apparatus of a cell and, thus, causing the cell cycle arrest are called mitotic poisons. Mitosis is a series of biological events in a cell that has to pass many checkpoints. Changes in these control mechanisms caused by mutations in corresponding genes lead to cancer development and progression, which results in uncontrolled cell division [4]. Therefore, in order to stop such undesired cancer cell division, mitotic poisons can be applied. The largest subgroup of mitotic poisons are microtubule inhibitors. In mitosis, microtubule fibers form a mitotic spindle, which is essential for correct distribution of genetic information. In a case when the mitotic spindle is not formed, chromosomes cannot be equally distributed, which generally leads to cell death. However, at some occasions, cells can also bypass the step of cytokinesis and leave mitosis as tetraploid ones. What could be quite surprising, though, is the fact that mitotic poisons do not target only microtubules but also a number of other proteins, namely various kinases and kinesins, all of which will be discussed further in this review article.

## 2. Microtubule Inhibitors

Microtubules are polymeric protein fibers, the basic element of which is a dimer of α- and β-tubulin. These dimers interconnect and create protofilaments. Thirteen protofilaments form a hollow fiber of microtubules in human cells. At the so-called minus end of microtubules, which is connected to the nucleation center, there is an α-tubulin subunit, while the β-tubulin subunit is located at the opposite side of the fiber, the so-called plus end. The minus end of the microtubule fiber is stabilized by a cap formed by a γ-tubulin subunit [5]. The basic and key property of these polymers is the so-called dynamic instability of microtubules [6]. The microtubule fibers permanently grow and decay, which is an essential feature of their biological functions, especially of the function of the mitotic spindle. Tubulin dimers can be disconnected from or attached to both ends of the fiber. Interestingly, much faster changes occur at the plus end of the fiber, i.e., on the opposite side to the nucleation center [7]. Microtubules are essential for maintaining the cell shape, polarity, migration and division [5]. Furthermore, they are utilized by protein motors kinesins and dyneins to transport molecules inside a cell [8].

In addition to the α and β tubulin isoforms, other structurally similar proteins also belong to the tubulin superfamily creating six subgroups, α to ζ. Besides the α and β tubulin, the third most studied one is γ-tubulin, which is located in the nucleation center of microtubules and is involved in centrosome and basal bodies formation [9,10]. The role of other discovered tubulins has not been fully deciphered, yet. Some of these subgroups, e.g., ε- and δ-tubulin, are involved in cilia function, since a connection with microtubule triplets of basal bodies has been proven [11,12]. Further, the structural and functional diversity of tubulins is provided also by numerous posttranslational modifications (PTMs), for example, by tubulin acetylation, which is one of the most common one. PTMs affect the microtubule structure, function, stability and ability to bind other proteins. For more details on tubulin‘s PTM, see a comprehensive review by Song and Brady (2015) [13]. 

**Figure 1:**
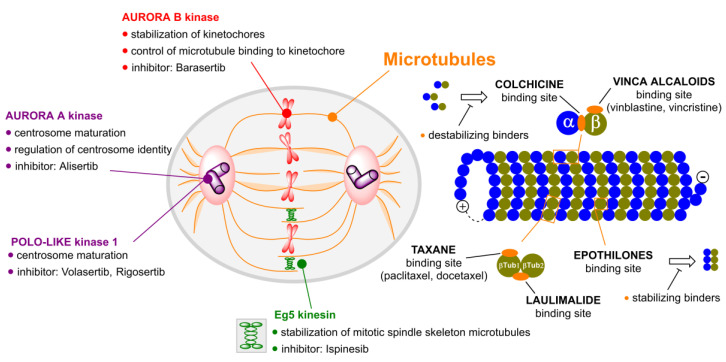
Mitotic poisons and their targets, cellular functions of polo-like kinase, aurora kinases A and B and kinesins, inhibitors of these proteins, inhibitors of tubulin and their binding sites.

Substances that target microtubules (Figure 1) can be divided into tubulin polymer stabilizers (e.g., taxanes) and compounds which destabilize the tubulin fibers and cause their disintegration (e.g., vinca alkaloids). 

Tubulin inhibitors can be also classified according to the site at which they bind to tubulin. There are five major binding sites to which tubulin inhibitors bind: the binding site for colchicine, taxanes, laulimalide, epothilones and vinca alkaloids. Multiple clinical trials (Table 1) have shown that some of them lack a therapeutic window and are dose-limited by toxicities to tissues. However, given their high affinities and potencies, we contend that mitotic inhibitors can find clinical utility if chemically refashioned with cancer-specific moieties.

In the last few years, several molecules have been developed that selectively target the mitotic apparatus to prevent cell duplication or enhance cell death. Here, we summarize the preclinical and available clinical data on the microtubule targeting agents presented in this review article. In Table 2, the clinical trials on targeted mitotic inhibitors are displayed, from both natural and synthetic origin, used as single agents.

### 2.1. Colchicine

**Figure 2:**
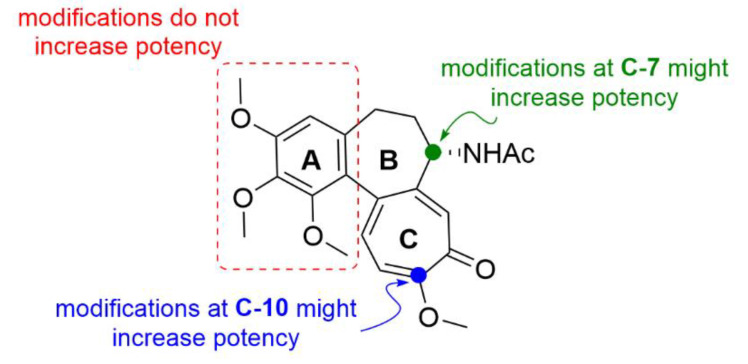
Chemical structure of colchicine **1** and suitable positions for its derivatization.

Colchicine (**1**; Figure 2) is a tricyclic tropolone alkaloid naturally occurring in all parts of the *Colchicum autumnale* plant. It was first isolated in 1820, however, the *Colchicum* extracts have been used to treat arthritis since 1500 B.C. [15,16]. The effect of compound **1** on mitosis was first described by Italian scientist B. Pernice in 1889. In his experiment, he observed division of gastrointestinal cells of two dogs treated with compound **1** and besides other things, he described only a rare occurrence of late stages of the cell division [17]. The most studied property of compound **1** is its ability to bind to tubulin dimers. The binding site of compound **1** is located at the interface of two tubulin subunits in a dimer [18]. The tubulin dimer bound to compound **1** is still able to attach to the growing microtubule fiber, but additional tubulin dimers cannot be attached to this modified/terminal dimer, which results in microtubule growth inhibition. At substoichiometric concentrations of compound **1** in relation to cellular tubulin, compound **1** stabilizes the microtubule fibers, whereas at stoichiometric or higher concentrations, it induces microtubule depolymerization [19]. Thus, compound **1** stops the cell cycle in the G2/M phase [20], which can be utilized in clinics.

**Figure 3:**
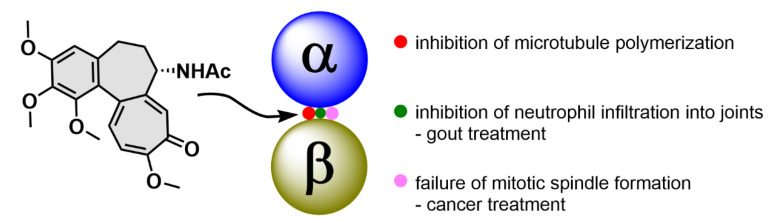
Tubulin binds a specific site between α and β subunit of a tubulin dimer, which causes inhibition of further microtubule polymerization. This phenomenon can be utilized in the treatment of gout and cancer.

Compound **1** has exerted therapeutic effects in a range of various diseases. One of the first applications of compound **1** was treatment of gout attacks. The mechanism of action of compound **1** in this disease also lies in microtubule fibers stabilization, which results in disruption of vesicular transport. Among other events, the vesicular transport is essential for cytokine secretion. When the transport is disrupted, basic cell functions such as chemotaxis, adhesion and phagocytosis by neutrophils are limited [21]. In addition, microtubules also play a significant role in the neutrophil ability to deform and, thereby, pass through the capillary wall into tissues (diapedesis, extravasation). Since compound **1** negatively acts on this neutrophil ability, neutrophils cannot get directly into the joints and cannot elicit the immune response [22]. Probably, by a very similar mechanism as in gout attacks, compound **1** also helps to prevent acute attacks of an autoimmune inflammatory disease, familiar Mediterranean fever [23]. As aforementioned, microtubules play an essential role in cell division, because they form the mitotic spindle. By inhibition of its formation, it is possible to stop the cell division and consequently induce cell death, which can be utilized especially in cancer treatment (Figure 3). Nevertheless, the possible use of compound **1** in tumor chemotherapy is highly limited because of a very low selectivity of compound **1** for cancer cells, which results in a rather low therapeutic index [24].

One of the possibilities of how to use compound **1** in cancer treatment is a so-called combination therapy, which, however, stays yet more at the theoretical level. Bhattacharya et al. (2016) reported a study on this topic, in which they found that at clinically approved concentrations (2.5 nM) of compound **1**, it neither exhibited signs of cytotoxicity, nor stopped the cell cycle in the G2/M phase in A549 cells derived from lung carcinoma. What they observed though, was reactive oxygen species (ROS)-mediated induction of autophagy and cell senescence. Moreover, the autophagy inhibitor 3-methyladenine was able to increase the cytotoxicity of compound **1** and switch cell senescence to apoptosis. At the same time, such a combination of compounds demonstrated lower cytotoxicity in noncancerous cells of primary lung fibroblasts WI-38 than of their cancerous counterparts. Therefore, the combination of compound **1** and 3-methyladenine could be an effective tool to combat lung cancer [25].

**Figure 4:**
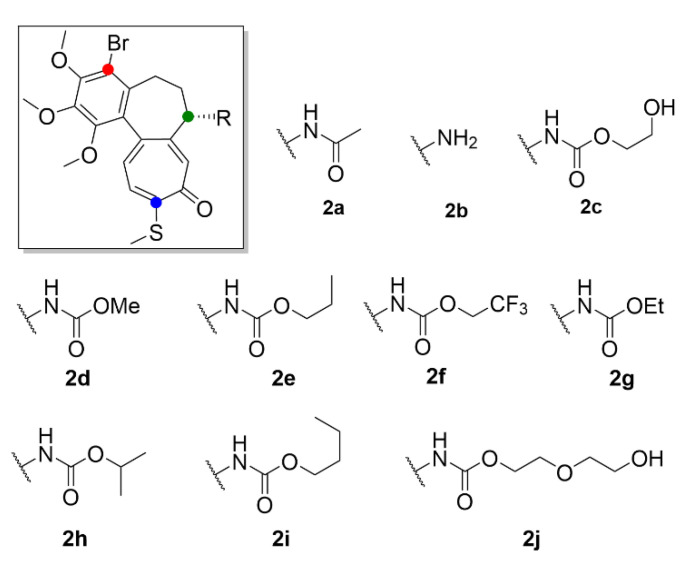
Colchicine derivatives **2a**–**2j**, adapted from [26].

Another approach on how to increase the therapeutic index of compound **1**, is its derivatization. It may provide a more selective and less toxic compound potentially applicable in clinical practice. Majcher et al. (2018) synthesized nine triple-modified derivatives of compound **1** (compounds **2a**–**2j**, Figure 4). Their activity (Table 3) was tested in four human cancer cell lines (A549, MCF-7, LoVo, LoVo/DX) and compared to the activity of commonly used cytostatic chemotherapeutics doxorubicin and cisplatin. Compounds **2a**–**2j** structurally differ from compound **1** by a substitution with bromine on the benzene ring, by replacement of the methoxy group with a thiomethyl group on the tropolone ring, and also by substitutions on the amide group while maintaining the stereochemistry. Practically all these derivatives (**2a**–**2j**) of compound **1** exhibited enhanced cytotoxicity in comparison to the commonly used cytostatics, and most of them showed higher toxicity than compound **1**. Four of the derivatives displayed IC_50_ values (Table 3) in the nanomolar range and were also the least toxic for noncancerous primary mouse fibroblasts [26]. It was previously confirmed that thiocolchicines have higher molecular stability and bind to tubulin more rapidly than compound **1**, whereas the strength of the bond is almost identical [27]. Similarly, Yasobu et al. (2011) reported that halogenation of the benzene ring of compound **1** results in a significant increase in cytotoxicity and selectivity for cancer cells [28].

**Figure 5:**
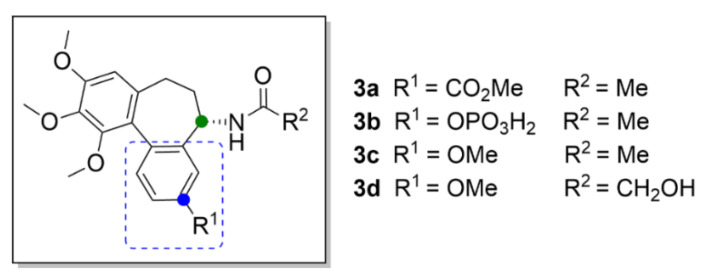
Allocolchicine derivatives, adapted from [29,30,31].

Most probably, the key structural motif of compound **1** responsible for the mechanism of action is the benzene ring with three methoxy groups. Even small structural variations in this key motif, such as an isomeric shift of one of the methoxy groups to a different position at the aromatic ring, completely change its mechanism of action, as shown, for example, at (S)-3,8,9,10-tetramethoxyallocolchicine (**3c**, Figure 5). Compound **3c** is a derivative of allocolchicine (**3a**, Figure 5), a molecule structurally similar to compound **1**, which has a six-membered ring instead of the seven-membered tropolone ring. Compound **3c** induces ROS production resulting in autophagy of human cells derived from pancreatic carcinoma and unlike compound **1**, it does not affect microtubule polymerization.

Compound **3a** has recently become more attractive for novel drug development than compound **1**, since it is easier to modify. Transformation of the tropolone backbone provides additional possibilities for efficient derivatization. Compound **3a** also maintains the anticancer activity and, thus, it is suitable for development of novel anticancer drugs [31]. Great attention was paid also to the discovery of an allocolchicine derivative ZD6126 (**3b**, Figure 5) applicable in the form of a water-soluble phosphate prodrug, which is converted in vivo by serum phosphatases to the active compound. It is a similar substance to compound **1**, which has a benzene ring with a phosphate group attached instead of the tropolone ring. Compound **3b** is a very potent inhibitor of angiogenesis causing also tumor necrosis [30]. This derivative was included in clinical trials, which were, however, discontinued in the second phase, based on the occurrence of severe side effects, such as cardiotoxicity, at pharmacologically relevant doses [32].

**Figure 6:**
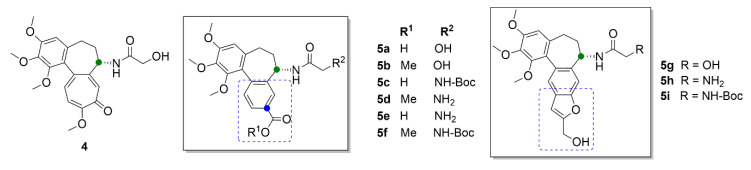
Colchifoline and its derivatives, adapted from [31].

Gracheva et al. (2018) synthesized bifunctional derivatives of compound **3a** (compounds **5a**–**5i**, Figure 6), the structure of which is based on the metabolite of compound **1**, colchifoline (**4**, Figure 6), which includes a hydroxyacetamide group instead of an acetamide group of compound **1**. Compound **4** binds tubulin with higher affinity than compound **1** and, thus, it also exhibits greater anticancer activity. This potency has been later explained as being caused by the hydroxy group in the structure. At the same time, it has been found that the hydrophobic group on the tropolone or benzene ring in compounds **4** and **3a**, respectively, also plays an important role in enhanced anticancer effect. An opposite effect exhibited derivatives with a free carboxy group, for which manifold lower anticancer activity was demonstrated in vitro in T3M4, MiaPaCa-2 and PANC-1 cell lines derived from pancreatic carcinoma. In the same cell lines, anticancer activity was also decreased when primary amines were present in the structure [31].

**Figure 7:**
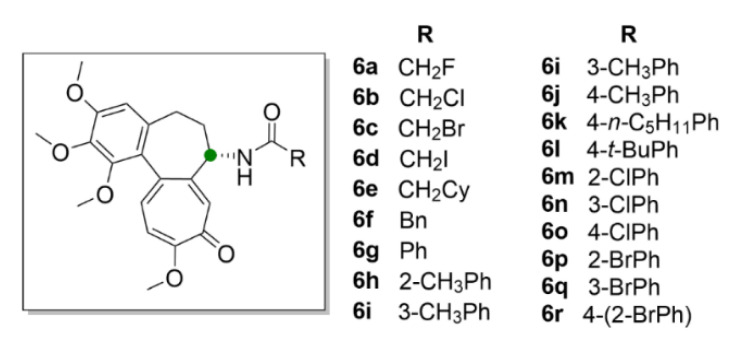
Colchicine derivatives substituted at the amide group, adapted from [33].

Large space for derivatization of compound **1** offers the seven-membered middle ring with the acetamide group. Marzo-Mas et al. (2018) synthesized haloacetyl and haloaroyl derivatives of compound **1** (compounds **6a**–**6r**, Figure 7), the biological activity of which was determined in three cancer cell lines, HT-29, MCF-7 and A549, and compared to a transformed noncancerous cell line, HEK-293. All of the compound **1** derivatives showed antiproliferative effects at nanomolar concentrations and most of them were more potent than the parental compound. At the same time, it was confirmed that all of these derivatives bound to the same β-tubulin-binding site as compound **1** and inhibited microtubule polymerization in the same manner as compound **1** [33].

Chlorbenzoyl (**6m**–**6o**) and brombenzoyl (**6p**–**6r**) derivatives of compound **1** exhibited the most pronounced effect on the cell cycle, which was arrested in the G2/M phase already at 20 nM concentrations, another derivative (**6c**) bearing a bromacetyl moiety at 15 nM concentration. Moreover, by this study, it was proved that these derivatives have a demonstrable effect on the regulation of the transcription factor c-Myc, human telomerase reverse transcriptase (hTERT) and the vascular endothelial growth factor (VEGF) oncogenes, via decreasing their expression. These genes also play a significant role in carcinogenesis and their overexpression is associated with a poor prognosis of cancer [33].

**Figure 8:**
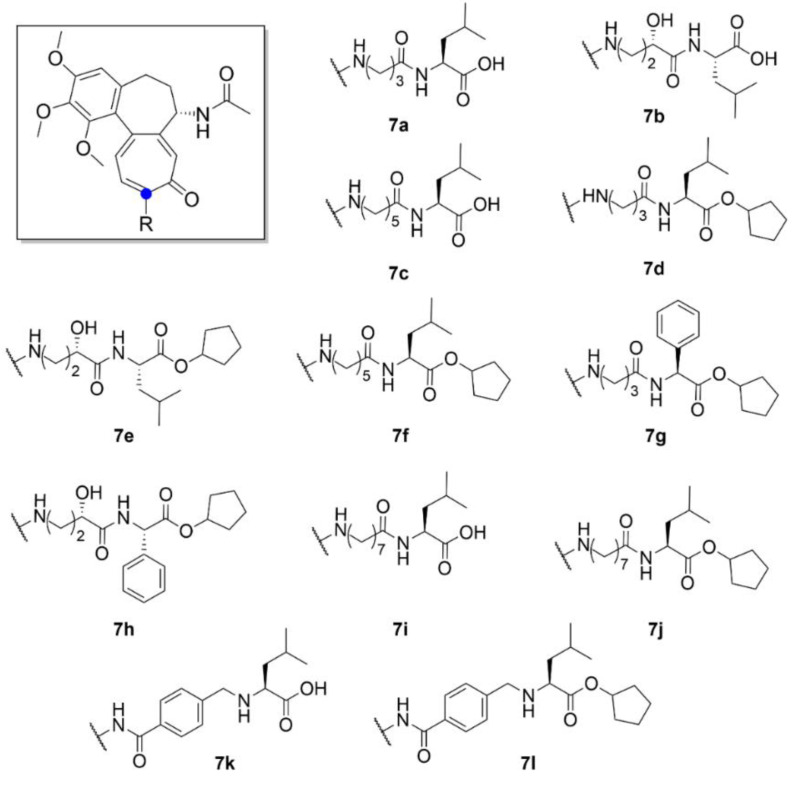
Colchicine derivatives substituted at the amine group, adapted from [35].

Besides cancer treatment, compound **1** derivatives could also find an application in the treatment of certain viral diseases. It has been found that the life cycle of some vector-borne flaviviruses is affected by the microtubules of a host cell. For example, Zika virus causes dramatic changes in the distribution of host cell microtubules. These then form a cage-like structure around the replication apparatus of the virus and thus stabilize it [34]. Richter et al. (2019) synthesized a series of derivatives (compounds **7a**–**7l**, Figure 8) of compound **1** with different side chains bound to the amine at C-7 position. Two of the thirteen derivatives exhibited almost similar antiviral activity as that of compound **1**, but while being far less toxic than compound **1** (ten times lower IC_50_ after 72 h). The molecular docking studies showed that these derivatives form less interactions with tubulin than compound **1** and, thus, are weaker tubulin binders. However, tubulin binding is apparently not the only mechanism of compound **1** antiviral activity, since cytotoxicity, antiviral activity and tubulin binding ability did not fully correlate. Given the insufficiently significant differences in the cytotoxicity of cleaved and uncleaved derivatives, it is clear that the selective motif for human carboxylesterase is not essential for the biological effect of these derivatives [35].

**Figure 9:**
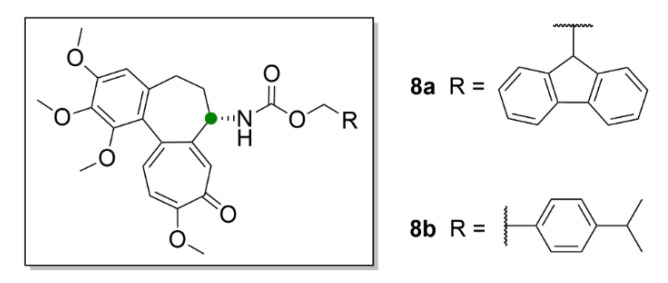
Colchicine derivatives with a carbamate group, adapted from [36].

Another possible utilization of compound **1** and its derivatives in medicine could be in transplantations, since the antiproliferative effect of compound **1** could be advantageously employed to suppress cellular immunity. Choi et al. (2019) synthesized derivatives of compound **1** (**8a** and **8b**, Figure 9), which have a carbamate group on C-7 instead of the acetamide group.

Compound **8a** and **8b** inhibited T-cell lymphocytes at nontoxic concentrations in mouse models and caused longer survival rate of the transplanted grafts [36]. Nevertheless, for further research of compound **1**, but also due to its use in modern medicine, it is necessary to optimize the possibilities of its industrial production.

Compound **1** is currently obtained only by extraction from seeds of *Colchicum autumnale* and seeds and tubers of *Gloriosa superba*. Although the first syntheses of compound **1** were performed already in 1959, till now, no commercially profitable way of its synthesis has been found [37,38]. One of the newest synthetic approaches is an enantioselective synthesis according to Chem et al. (2017). They synthesized compound **1** in nine steps with an overall yield of 9.2%. The starting compound for this synthesis is a commercially available 2,3,4-trimethoxybenzaldehyde [39]. However, more facile processing and higher yields are the reason for current use of extraction methods in industrial processing, the most often utilized of which are the soxhlet and solid-to-liquid extraction methods [40]. Nonetheless, even these methods do not provide sufficient yields which would be required for clinical use, therefore, novel methods for compound **1** production are being considered, for example, biotechnology from plant tissue cultures [41]. A novel interesting method for compound **1** production is the use of biorhizoma. It is an asexually produced root system grown in vitro. The *Gloriosa* biorhizoma produces compound **1** constantly regardless of the weather or the seasons of the year. However, for a more cost-effective production of compound **1** by this new technology and its massive use, it is necessary to understand the molecular mechanisms in the biorhizoma and the biosynthetic pathway of compound **1** in more detail and to use molecular engineering methods to start overproduction of this alkaloid in the bioreactor [42].

### 2.2. Taxanes

**Figure 10:**
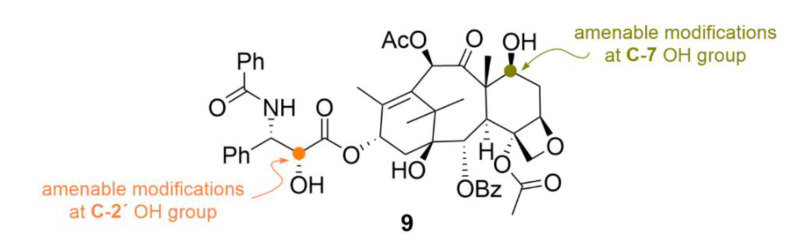
Chemical structure of paclitaxel **9** with allowed positions for its conjugation.

Another group of substances which bind to microtubules, taxanes, has found a broad use in cancer treatment. Taxanes are diterpenes of natural origin which were named due to the Latin name of a Mediterranean yew—*Taxus brevifolia*, from which they are isolated. The most well-known taxane is paclitaxel (compound **9**, Figure 10), which was first isolated in 1971 as a part of a project of American National Cancer Institute seeking for novel anticancer drugs [43]. In contrast to compound **1**, compound **9** binds preferentially to the microtubule fiber rather than to the free tubulin dimer [44]. What is interesting is the fact that the very nature of the compound **9** effect also differs from other microtubule inhibitors. De facto, compound **9** should not even be referred to as an inhibitor since it, on the contrary, causes a higher production of tubulin and microtubules, which are very stable, and that is what causes their disrupted functionality [45]. Compound **9** is used primarily to treat ovarian and breast cancer; it is also often administered in a combination with other cytostatics—for details, see below [46].

Though, what impedes more massive clinical use of compound **9** is its low water solubility. Therefore, it is necessary to search for novel matrices which would increase its water solubility. So far, the most well-known formulation of compound **9** is marketed with a trade name Taxol^®^. This slightly yellowish viscous solution uses Cremophor^®^ EL for improved water solubility of compound **9**, which, however, causes hypersensitive reactions and, thus, unpleasant side effects after Taxol^®^ administration [47]. Thanks to development of nanocarriers, this problem is currently being eliminated. In the form of polymeric nanoparticles, albumin-bound compound **9** is currently marketed with a trade name Abraxane^®^ [48]. Recently, nanoparticles with poly(l-Lys) and l-Cys from modified chitosan were designed for incorporation of compound **9** in their hydrophobic core. In vitro studies have confirmed increased penetration of this drug into cells derived from colon adenocarcinoma (Caco-2 cell line) when compared to Taxol^®^ itself. Regarding bioavailability, in pharmacological studies, compound **9** was five times more bioavailable than in Taxol^®^ formulation. In addition, lower toxicity and better distribution of this way administered compound **9** in tumors in mouse models compared to Taxol^®^ was confirmed [49].

Another way to enhance the water solubility of compound **9**, is the use of liposomes, as in the case of Lipusu^®^ agent, or encapsulation into polymer micelles as, for example, in the Nanoxel^®^ agent [50,51]. A major limitation of broader clinical use of compound **9** is also the fact that despite a successful outcome after the first series of chemotherapy with this drug, most patients experience a relapse of the disease and an increase in cancer cell resistance to the drug. There are several mechanisms of such resistance, the main ones being the expression of genes leading to multiple drug resistance, for example, efflux pumps, and also changes in the microtubule system [52].

**Figure 11:**
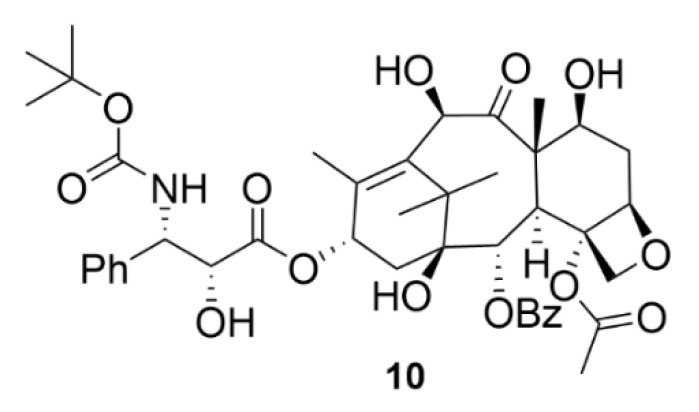
Chemical structure of docetaxel.

In addition to compound **9**, there is another currently used drug from the taxane group, namely, docetaxel (compound **10**, Figure 11), marketed as Taxotere^®^. Compound **10** is a semisynthetic compound, which is formed by esterification of 10-deacetylbaccatine III, which naturally occurs in the needles of *Taxus baccata*. Compound **10** finds its use in the treatment of many types of solid tumors, such as breast, ovarian, lung or head and neck tumors [53]. Although the chemical and biological nature of compounds **9** and **10** is to a large extent similar, clearly, the development of resistance to each compound occurs by a different mechanism. It is clinically confirmed that compound **10** is effective against tumors resistant to compound **9** [54].

One way to improve the clinical properties of taxanes is their derivatization. There is plenty of compound **9** derivatives, reviewed in detail by Leonelli et al. (2008) [55]. The first derivative of compound **9** specifically targeting tumors was synthesized in 1999. It utilizes a specific short peptide recognizing the bombesin receptor, which is linked to compound **9** by using a polyethylene glycol linker (PEG). At the same time, PEG ensures improved water solubility of this conjugate, which has higher cytotoxicity in cells from lung carcinoma (NCI-H1299 cell line) with the IC_50_ value ten times lower than that of compound **9** [56]. This general principle of compound **9** conjugation with molecules increasing water solubility and targeting into tumors is commonly used also today. At the same basis, synthesis of a derivative of compound **9** modified with dextran for enhanced water solubility and by folate for targeting the folate receptor was performed, since the folate receptor is being overexpressed in some types of tumors. This conjugate of compound **9** was approximately fourteen times more soluble than Taxol^®^ and its cytotoxicity in cells derived from oral cavity carcinoma (KB cell line) overexpressing the folate receptors was three times higher [57].

An interesting solution to ensure targeting specificity is conjugation of compound **9** with a nucleoline aptamer. Aptamers are short RNA or DNA oligomers or peptides which specifically bind cell structures with a very high affinity, reviewed in detail by Darmostuk et al. (2015) [58]. Their big advantage is low immunogenicity and low probability of resistance development. The nucleoline protein, which is normally localized in the cell nucleus, is also expressed on the cytoplasmic membrane in some types of cancer cells. Compound **9** modified by the nucleoline aptamer exhibits higher cancer cell selectivity, lower overall toxicity and high anticancer activity in ovarian cancer [59].

The leading line in taxane research, and particularly in research of compound **9**, is the development of combinatorial therapy, i.e., a synergistic effect of multiple drugs together. Since some cancer cells are resistant to compound **9**, which acts as an apoptosis inducer, recently, a study has been published on how to overcome this issue. The study describes a co-administration of a liposomal formulation of compound **9** and siRNA (short/small interfering RNA) for a gene encoding the survivin protein. Such a combination was chosen since survivin belongs to anti-apoptotic proteins which are commonly overexpressed in tumor cells. The interference of siRNA with mRNA for survivin results in decreased expression of this anti-apoptotic protein, so that the cancer cells are more sensitive to compound **9** leading to cell death. The results of this study confirmed the theoretical findings; the liposomes can effectively deliver both molecules into cells derived from lung carcinoma (NCI-H460 cell line), in which the IC_50_ was three and half times lower than that for compound **9** administered alone. The main advantage of this synergistic action is a possible administration of compound **9** at lower doses [60]. Another example of utilizing compound **9** in a combinatorial approach for cancer treatment is its administration with ferulic acid or its derivatives. Ferulic acid is an inhibitor of efflux pumps, such as P-glycoprotein, which is responsible for multidrug resistance of cancer cells leading to decreased outcome when using compound **9** alone. Administration of ferulic acid and/or its derivatives in a combination with compound **9** increases its biological availability and, therefore, it can increase the treatment efficiency of tumors with P-glycoprotein overexpression [61].

The evidence of extensive research in the field of combinatorial therapy with compound **9** together with other drugs is given by the number of clinical trials. The website https://www.clinicaltrials.gov/ (15.9. 2020) shows 2007 results for the term “paclitaxel combination”, from which 30 studies were in the fourth phase of clinical trials and 328 in the third phase. In the clinical practice, compound **9** is quite commonly used in a combination with carboplatin. These two drugs forming a sort of a tandem are known as CarboTaxol. CarboTaxol is used to treat ovarian, cervical and non-small cell lung carcinoma. At the same time, in clinical trials, these two compounds have started to be combined with biological therapy utilizing monoclonal antibodies, such as bevacizumab, an antibody targeted against VEGF. This drug combination exhibits very positive therapy outcomes [62,63,64].

The production of compound **9** remains a persisting problem. The industrial production of compound **9** is still carried out by the extraction and purification from the inner bark of *Taxus*. This is not only a very extremely ecologically demanding process but also the overall yields of compound **9** are very low [65]. Total synthesis of compound **9** has been a great challenge for scientists from all over the world for long time. However, in 1994, two first total syntheses were reported almost simultaneously. Holton et al. (1994) synthesized compound **9** in forty-six linear steps from patcholene oxide [66]. Nicolau et al. (1994) managed the total synthesis of compound **9** in only forty steps in three separate phases using mucic acid as a starting compound [67]. In the following years, a number of other procedures for total synthesis of compound **9** were published [68,69,70,71,72,73,74]. Unfortunately, none of these can be applied for its commercially profitable production due to low yields [65].

However, a commercial use has found semisynthesis of compound **9** developed by Bristol-Myers Squibb Company using 10-deacetylbaccatin III as a precursor. It is de facto compound **9** lacking the side chain at C-13 and acetate at C-10. This precursor can be gained by an enzymatic reaction of different taxanes obtained from the biomass and, then, it is connected to an artificially synthesized side chain. The whole semisynthesis, both getting the precursor and synthesizing the side chain runs via biocatalysis by microbial enzymes [75]. Similarly, a more modern semisynthetic procedure by the Natural Pharmaceuticals Company utilizes a mixture of structurally similar taxanes. This semisynthesis is based on the isolation of taxanes with different substituents on the terminal amide group of the side chain in comparison to compound **9**, which has a phenyl group in this position. This semisynthesis uses a conversion of the amide to amine by using Swartz agent followed by benzoylation. Such a method for compound **9** production is very fast and effective. Unfortunately, it still requires isolation from biological material of the yew, which grows very slowly (3–4 years of regeneration after harvesting) and, therefore, it is ecologically very demanding and unfavorable [76]. Unfortunately, neither a biotechnological way to produce compound **9** in fungi has found its way to the industrial practice, yet, although it has been extensively researched and discussed for decades. So far, many compound-**9**-producing fungal strains have been discovered, the most effective of which is *Aspergillus fumigatus* KU-837249, which is able to produce 1.6 g of compound **9** per 1 L of medium. By the proper genetic engineering of fungal DNA, for example, by changes resulting in an increased expression of the *dbat* gene responsible for compound **9** production, its commercial use could be enabled [77]. However, another key factor is also the cultivation itself. By proper modifications of the media and cultivation conditions, it should be also possible to increase the production of compound **9**; a positive role for its total yield was reported for cultivation of *Aspergillus fumigatus* at the darkness [78].

### 2.3. Laulimalide

**Figure 12:**
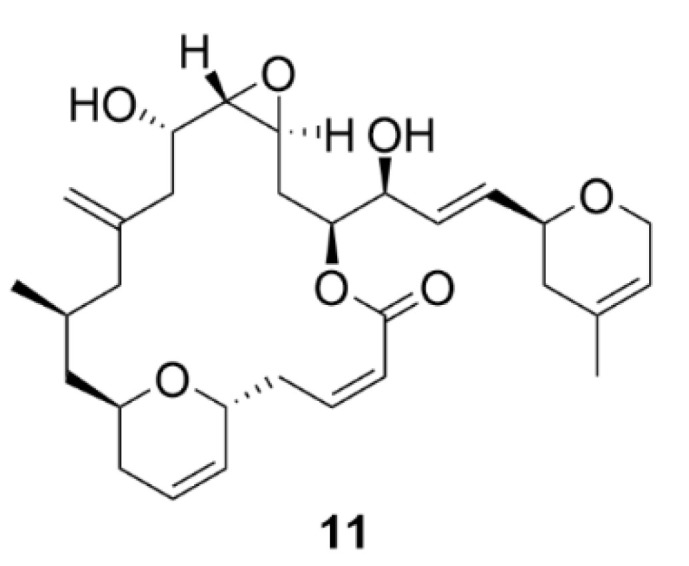
Chemical structure of laulimalide.

Another agent affecting microtubule polymerization is laulimalide (**11**, Figure 12). Compound **11** is a natural compound first isolated in 1999 together with a structurally related isolaulimalide from the marine sponge *Cacospongia mycofijiensis* [79]. Compound **11** binds to tubulin at a unique site between the two β-subunits of the tubulin in the microtubule fiber [80,81]. In theory, this unique binding site could be advantageously utilized in treatment of the taxane-resistant tumors, or a synergistic effect could be employed when co-administrated with taxanes [82,83]. Another big advantage is also the fact that compound **11** is not a P-glycoprotein substrate [82]. Just as taxanes, compound **11** causes increased polymerization and stabilization of microtubules resulting in inhibition of cell proliferation of different cancer cell lines in vitro. Quite disappointingly though, in vivo experiments have shown manifold lower inhibitory activity of cancer cell proliferation and even worse, high systemic toxicity [84]. Moreover, it has been demonstrated that compound **11** is quite unstable in vivo. This issue could be easily overcome by derivatization, nevertheless, such derivatives of compound **11** possess decreased biological activity than the parental compound [85,86,87,88,89]. For these reasons, compound **11** has not yet found its application in the clinics, although the interest in this compound was great after its discovery, the evidence of which is documented by many of its available synthetic approaches [90,91,92,93,94,95,96,97,98,99,100]. Therefore, compound **11** is now used only by the scientific community for research purposes to reveal microtubule-associated processes [101].

### 2.4. Peloruside A

**Figure 13:**
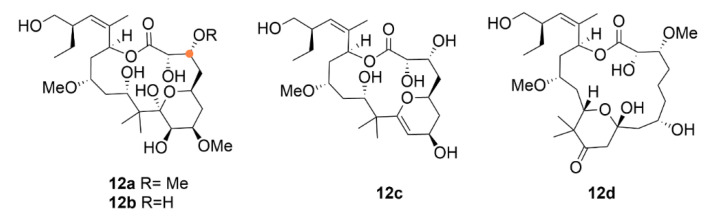
Chemical structure of pelorusid A.

Pelorusid A (**12a**, Figure 13) is a macrocyclic secondary metabolite from a marine sponge of *Mycale sp.* binding-site-wise related to compound **11**. Compound **12a**, which binds to the exact same place on tubulin as compound **11** does [102], classified as a macrolide, was discovered and isolated in 2000 [103]. Similar to compound **9**, compound **12a** induces polymerization of microtubules, arrests the cell cycle in the G2/M phase and triggers cell death by apoptosis [104]. In the mouse model, it exhibited very big anticancer potency. Compound **12a** was even more effective than the commonly used therapeutic (compound **9**) treatment of non-small cell lung cancer xenografts (H460 cell line). At a dose of 5 mg·kg^−1^, 84% inhibition of tumor growth was achieved, whereas compound **9** (8 mg·kg^−1^) inhibited tumor growth only down to 50%. In addition, for A549 cell xenografts, compound **12a** displayed better results than compound **9** (44% inhibition at the dose of 16 mg·kg^−1^); the tumor growth inhibition ranged between 51% and 74% depending on the type of dosing (5–15 mg·kg^−1^) [105].

In addition to inducing apoptosis, compound **12a** acts also by a different mechanism, causing a so-called accelerated senescence of tumor cells. This process is similar to the replicative senescence that is a definitive arrest of cell growth and division caused by telomere shortening. Cells usually stop their growth in the G1 phase, but typically DNA disrupting chemotherapeutics and mitotic poisons cause the cell cycle arrest in the G2/M phase, summarized in Gewirtz et al. (2008) [106]. The ability of compound **12a** (IC_50_ = 10 nM) to induce accelerated senescence was demonstrated in breast cancer cells (MCF-7 cell line) proven by the quantification of β-galactosidase, which is associated with cell senescence, but this ability has been significantly lower than that of all the control compounds including compound **9** (IC_50_ = 6 nM**)**. The same percentage of senescence was proved for IC_25_ of compound **9** and IC_70_ of compound **12a**. The ability to cause accelerated senescence is strongly concentration-dependent, and it grows with concentration and toxicity. That is why it is not possible to exactly determine what the contribution of accelerated senescence to the overall anticancer activity of compound **12a** is. For that, it is necessary to further study the complex mechanism of action of this compound [107]. 

Besides the direct cytotoxicity, compound **12a** has exerted great results also in the area of angiogenesis inhibition with the IC_50_ of 1.4 nM in umbilical vein endothelial cells. At the same time, compound **12a** inhibited migration of the endothelial cells after injury (inhibition of wound healing after 18 h was achieved at IC_50_ of 6.3 nM) and cell organization into the capillary-like tubes was assessed at IC_50_ = 4.5 nM after 16 h of cultivation in Matrigel^TM^ [108]. Besides the anticancer activity, compound **12a** shows also promising results in treatment of neurodegenerative diseases, since it is able to stabilize microtubules in the damaged neurons [109]. Extra to anticancer treatment, mitotic poisons can generally be used also for the treatment of immune disorders, inflammations, etc. Compound **12a** is no exception. For example, it was reported that compound **12a** delays the onset of autoimmune encephalomyelitis by inhibiting the proliferation and migration of immune cells into the central nervous system (CNS) [110,111].

After the discovery of compound **12a**, interest in the sponge *Mycale hentscheli* from which it was isolated has not ceased; it has been studied further and soon an analogue of compound **12a** has been discovered—peloruside B (**12b**). This molecule differs from compound **12a** only by small structural changes: presence of a hydroxy instead of a methoxy group. Although the mechanism of action of both substances is identical, i.e., cell cycle arrest in the G2/M phase, the IC_50_ values in cells from human myeloid leukemia (HL-60 cell line) differ quite significantly, i.e., 33 nM and 10 nM for compounds **12b** and **12a**, respectively. Besides compounds **12a** and **12b**, another two analogues have been discovered, peloruside C (**12c**) and D (**12d**). These two differ from **12a** and **12b** in the oxygenation level at the pyran ring, which leads to a decrease in the anticancer activity. Compound **12d** differs dramatically from all the other compounds by shifting the pyran ring due to which it completely loses the anticancer activity [112]. 

The reason why compound **12a** and **12b** have not been included in any clinical trial, yet, is their insufficient supply and inability to produce these compounds at high yields [113]. That is why, similarly to other aforementioned compounds, novel ways of their industrial production are being sought. Up to now, six total syntheses of compound **12a** have been reported [114,115,116,117,118,119]. At the same time, twenty novel derivatives, arisen either by total synthesis or semisynthetic approaches, have been presented, summarized in Brackovic et al. (2015) [120]. The most recent study on this topic was published by Chany et al. (2019), who synthesized four novel derivatives of compound **12a** by connecting four separate synthetic components. The main difference among the derivatives and compound **12a** is the absence of almost all methoxy groups in their structure. These structural changes represent a significant change in the anticancer activity of these derivatives when compared to the parental compound, the activity decreased from nanomolar to micromolar ranges [113].

### 2.5. Epothilones

**Figure 14:**
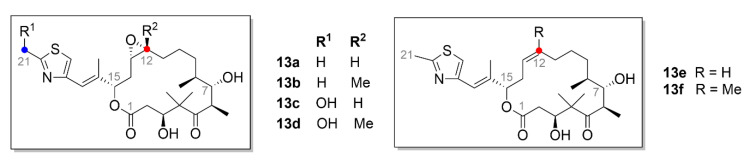
Chemical structures of epothilones.

Epothilones (Figure 14), which belong also to the microtubule inhibitors, are macrocyclic compounds isolated from the bacterium *Sorangium cellulosum* based on the 16-membered lactone ring. Six major epothilones, EpoA–EpoF, and more than thirty other related compounds have been identified, so far [121,122]. EpoA (**13a**, Figure 14) and EpoB (**13b**, Figure 14) are bacterial natural products, while EpoC (**13e**, Figure 14) and EpoD (**13f** Figure 14), which were discovered later, are their precursors and lack the epoxide group [123]. EpoE (**13c**, Figure 14) and EpoF (**13d**, Figure 14) are then formed by hydroxylation of the terminal C-21 methyl of compound **13a** or **13b**, respectively [122]. Epothilones have a similar mechanism of action as taxanes. Their main advantage when compared to compound **9** is enhanced water solubility, which is thirty times higher than that of taxanes, and also efficacy against taxane-resistant tumors [124]. The epothilone binding site in tubulin significantly overlaps the taxane binding site, therefore, epothilones compete in tubulin binding with compound **9** [121,125].

The first compound to reach clinical trials was compound **13b** marketed as patupilone. Compound **13b** is a very efficient inhibitor of the growth of ovarian cancer cells. Its cytotoxicity determined for different ovarian cancer cell lines exceeded that of compound **9** and ranges at nanomolar concentrations (hundredths to dozens) [126]. In the treatment of this type of cancer, one needs to take into account that epothilones, in addition to apoptosis, elicit also another type of cell death, autophagy [127]. It has recently been found that the cytotoxic effects of compound **13b** in ovarian cancer cells (SKOV-3 and OV-90 cell lines) could be increased by an autophagy inhibitor [128].

Sundry analogues and derivatives of compound **13b** are also being used in the clinical practice. One of them is ixabepilone (marketed as Ixempra^®^ in the USA), a derivative of compound **13b**, which is used to treat taxane-resistant metastatic breast cancer. This disease can be also treated by a combination therapy of compound **13b** with histone deacetylase inhibitor Vorinostat, which successfully passed the first phase of clinical trials [129]. Recently, ixabepilone was shown to be effective against chemoresistant triple-negative breast carcinoma treatment [130]. Combination therapy of ixabepilone co-administered with carboplatin may also be used to treat this and other types of breast cancer [131]. In clinical trials, the combination of ixabepilone with capecitabine, a commonly used chemotherapeutic for breast cancer treatment, was also successful [130].

One of the modern therapeutic approaches in cancer is specific tumor targeting, the aim of which is to circumvent problems with resistances to commonly used drugs and to limit side effects. One way of selective drug targeting to tumors is to conjugate a cytotoxic molecule with a molecule which interacts with cancer cell receptors. Such epothilone derivatives are still being researched and developed and one of them has even entered clinical trials. A derivative called BMS-753493 is an epothilone conjugated to folate, the receptors of which are overexpressed in cancer cells as aforementioned. This derivative passed the first phase of clinical trials, after which the next phase was discontinued since very limited anticancer activity was demonstrated [132]. A more recent attempt to target epothilone into tumors was carried out by Gaugaz et al. (2019), who synthesized a novel epothilone derivative with an incorporated *N*-(2-hydroxyethyl)-benzimidazole chain and described excellent antiproliferative properties of this derivative in nanomolar concentration range. Then, this highly potent conjugate with a peptide targeting the epidermal growth factor receptor (EGFR) was prepared. Interestingly, this conjugate retained the tubulin-binding activity despite the sterically very large interference into its structure, but its anticancer activity was significantly lower than that of the free epothilone derivative [133].

Epothilones are utilized not only in cancer treatment but also to treat other diseases. For example, compound **13f**, for its ability to penetrate the blood-brain barrier, can be used to treat various neurodegenerative diseases. In vivo, **13f** is able to slow down the progression of Alzheimer’s disease or frontotemporal dementia; these diseases are characterized by the nonfunctional tau protein and, therefore, are less stable microtubules [134]. Promising results exhibited epothilones in treatment of CNS injuries. Further, compound **13b** reduces scarring after rodent spinal cord injuries, because it prevents cell polarization and direct migration of the scar-forming fibroblasts. At the same time, it stabilizes microtubule polymerization in axonal projections and, therefore, axons can grow also in the injured environment [135]**.**

Epothilones are now produced by a biotechnological process from the bacterium *Sorangium cellulosum*. However, by utilizing commonly used methods, only low yields of epothilones (1 mg·L^−1^) are gained and also due to a difficult manipulation of the genome of this bacterium and its long doubling time, epothilones have not been largely used in clinical practice, yet [136,137]. One of the methods on how to increase production of these compounds is an incorporation of genome clusters for their production into another production organism. However, production of epothilones in other bacterial strains was not successful, e.g., in *Escherichia coli*, or in *Streptomyces coelicolor* [138,139]. Probably because of the bacteriostatic actions of epothilones, their yields in these bacterial strains are very low, e.g., in the case of *S. coelicolor* only 50–100 µg·L^−1^ [140]. Better results were obtained by production in a bacterium similar to the natural production strain, *Myxococcus xanthus*, the growth of which is manifold faster. After the growth condition optimization, the overall yield was augmented up to 23 mg of the compound per 1 L of medium [141].

Another way to achieve higher yields in epothilone production is a modification of the genetic information of the natural producer *Sorangium cellulosum*. For example, it is possible to incorporate genes for an increase in the secondary metabolite production, e.g., *vgb* gene which codes *Vitreoscilla* hemoglobin. This product ensures better transmission of oxygen within the cells, which has a positive impact on the epothilone production. In fact, *Sorangium cellulosum* grows in cell clumps and the utilization of oxygen during the mixing of the suspension is also quite limited. Insertion of this gene into the bacterial DNA really increased epothilone production by 58%. In addition, after insertion of the exogenic gene *epoF*, which codes a P450 oxidase catalyzing a creation of the epoxide bond, production of compound **13b** was increased by 122% in comparison to the strain lacking these genetic changes [142]. The most recent work on epothilone production is a study of Ye et al. (2019), who used transcription activator-like effectors transcription factors (TALE-TF) and CRISPR-based (clustered regularly interspaced short palindromic repeats) technologies of genetic engineering for this purpose. With use of these methods, a promotor for the genetic cluster of epothilone production was inserted into cells and the production of these compounds has significantly increased. By using the TALE-TF method, production of compounds **13b** and **13f** was augmented 2.89 and 1.12 times, respectively. Whereas by using CRISPR, the production of compounds **13b** and **13f** was 1.53 and 2.18 times higher, respectively, than that of the natural *Sporangium cellulosum* variant [136].

### 2.6. Vinca Alkaloids

**Figure 15:**
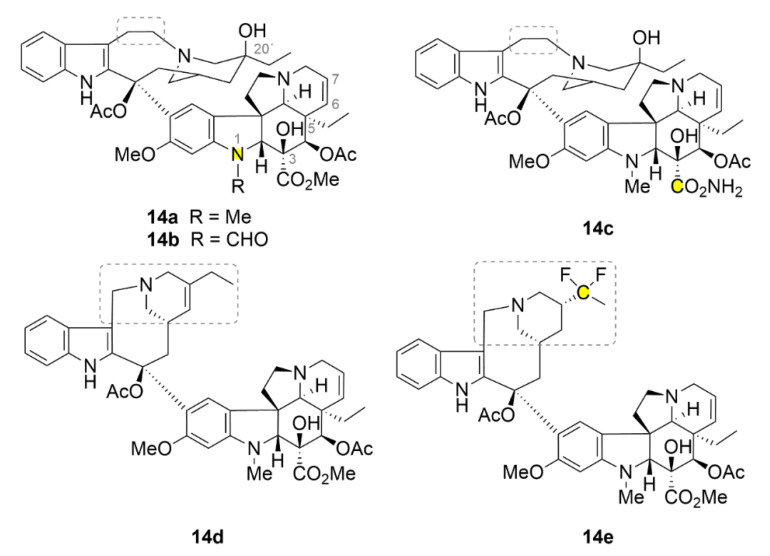
Chemical structures of vinca alkaloids.

The very first discovered compounds from the family of microtubule inhibitors are vinca alkaloids (Figure 15, compounds **14a**–**14e**). Their name is derived from *Vinca rosea* which is a former name of the *Catharanthus roseus*, an evergreen subshrub plant, from which they have been isolated in the 1950s of the 20th century. Currently, there are five compounds of the vinca alkaloids family available at the market: two natural compounds vinblastine (**14a**, Figure 15) and vincristine (**14b**, Figure 15), and three semisynthetic derivatives vindesine (**14c**, Figure 15) with an acetamide group on the cyclohexane ring, vinolerbine (**14d**, Figure 15) with similar structure to **14a** but with an ethyl tetrahydropyridine moiety and a shortened linker at the top of the molecule, and vinflunine (**14e**, Figure 15) as a difluoro derivative [143]. The exact binding site of these alkaloids on tubulin was evaluated more than fifty years after their discovery. Compound **14a** binds between two tubulin dimers and forms sort of a wedge that impedes further tubulin polymerization. As the only compound from the aforementioned microtubule inhibitors, it does not strictly bind only one tubulin dimer, but its binding site is divided between two of them. At low doses, vinca alkaloids stabilize the microtubule fibers by binding the growing end of the polymer and impeding the binding of another dimer. That could be used in anticancer therapy [144].

An interesting property of compound **14a** is its ability to form tubulin aggregates mostly of a spiral character. This property is caused by the fact that at higher doses, compound **14a** uses also other binding sites with usually lower binding affinity. After a series of conformational changes in tubulin, the microtubule fiber splits up. Subsequently, the various microtubule fibers are joined by means of compound **14a**, which, as aforementioned, preferably uses two different tubulin dimers for its binding, which do not have to be localized at the same fiber [144,145]. Compound **14a** has been the very first microtubule inhibitor used in clinical practice, namely in 1961 under the trade name Velban^®^. This drug has been widely used for the treatment of various types of tumors, for example, advanced breast cancer [146]. Nowadays, compound **14a** is still being used in the clinical practice and still undergoes various research trials. Most considered is its co-administration with other commonly used drugs, as seen, for example, in [147]. In 1963, another preparation containing vinca alkaloid **14b** has entered the drug market, namely, Oncovin^®^. Compound **14b** is now used in the treatment of acute lymphocytic and myeloid leukemia, non-Hodgkin’s lymphomas and other cancer diseases. Unfortunately, it causes some severe side effects associated mainly with peripheral neuropathy [143].

Similar to taxanes, resistance occurs during cancer treatment with vinca alkaloids. Today, this is being solved by co-administration with other drugs. Some combinations with vinca alkaloids are used as the main therapy for some types of cancer [143]. For B-cell lymphomas, the combination of compound **14b** with cyclophosphamide, doxorubicin and prednisone is primarily used. This doxorubicin-based chemotherapy protocol including cyclophosphamide, vincristine, and prednisone is known under an abbreviation CHOP (derived from: Cyclophosphamide, Hydroxydaunorubicin (doxorubicin), Oncovin (vincristine) and Prednisone). A chimeric monoclonal antibody against B-cell surface antigen rituximab is included to increase the efficacy of this combination [148,149]. The standard treatment for Hodgkin’s lymphomas is compound **14a** in combination with doxorubicin, bleomycin and decarbazine combined also with radiotherapy [150]. For stages IIIA and IIIB of non-small cell lung carcinoma, compound **14a** is used in a combination with cisplatin and radiotherapy (VCRT) [151]. Additionally, new possibilities for vinca alkaloid administration are being developed in order to increase their efficacy. Similar approaches to those described above for taxanes are used, these include encapsulation into liposomes [152], a use of nanoparticles [153] and others, see summarized in [143].

Similarly as for all the aforementioned compounds, also novel derivatives of vinca alkaloids are being developed. Leggans et al. (2013) synthesized series of compound **14a** derivatives modified with urea at C-20’ position. Although this position is important for tubulin binding and urea is quite a sterically demanding group, these derivatives possess a surprisingly high anticancer activity (IC_50_ in nanomolar ranges), which is in some cases more than ten times higher than that of the parental compound [154]. The urea motif seems to be very interesting for further research of vinca derivatives. It was found that compound **14b** with dimethylurea in C20 position binds stronger to the cytochrome P450 3A4 (CYP3A4) than to the cytochrome P450 3A5 (CYP3A5), which is mainly involved in the metabolism of compound **14b**. Patients overexpressing CYP3A5 experience unwanted neurotoxic side effects after compound **14b** treatment and, therefore, if CYP3A4 was used to metabolize this drug, therapy using compound **14b** could be more effective [155].

Surprisingly, the ethyl group at the C-5 position has a great impact on the activity of compound **14a**. Va et al. (2010) synthesized a series of derivatives, in which this ethyl group was substituted with different and sterically similar groups: H, Me, Pr, H, Me, Pr, CH=CH_2_, C≡CH, CH_2_OH and CHO. All of these derivatives possess lower anticancer activity compared to the natural compound **14a**, except for the methyl derivative, which has a similar activity as the parental compound [156]. The way to improve the biological properties of compound **14a** could be its conjugation to a carrier. By ensuring other mechanisms of internalization into cells, resistance in some types of cancer could be also overcome. This idea led to a synthesis of 17-desacetylvinblastine with oligo-arginines. Bánóczi et al. (2010) linked the C-3 (in original document numbered as C-16) carboxyl group of compound **14a** with L-Trp to its terminal amine and through its terminal carboxyl group they conjugated it to octa-, hexa- and tetra-arginines. All the derivatives retained the ability of binding to tubulin and also the antiproliferative activity, which was dependent on the number of arginines. In leukemic cells (HL-60 cell line), the greatest activity (IC_50_ ≈ 1.6 µM) was exhibited by the derivative with octa-arginines while the lowest one by the derivative with the shortest oligopeptide (IC_50_ ≈ 5 µM) [157].

### 2.7. Other Selected Tubulin-Binding Mitotic Poisons

**Figure 16:**
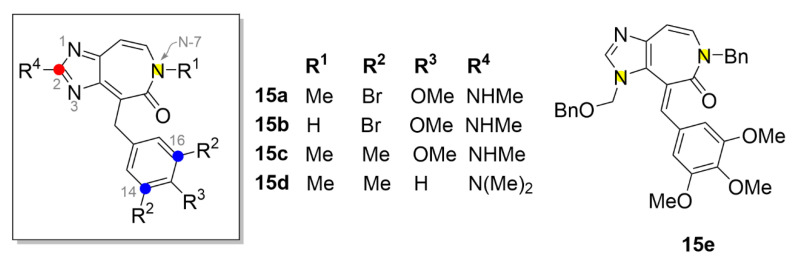
Chemical structures of ceratamines and their derivatives, adapted from [158,160,161].

Besides the aforementioned main groups of tubulin-binding mitotic poisons, there are also other compounds belonging among them sharing the same mechanism of action. One such example are heterocyclic alkaloids ceratamines A and B (compound **15a** and **15b**, respectively; Figure 16), which were isolated from the marine sponge *Pseudoceratina* sp. However, their cytotoxic activity is lower than that of the aforementioned compounds. For example, in the breast cancer cell line (MCF-7), the IC_50_ was determined to be 10 µM [158]. However, even despite such rather high inhibitory concentrations, they have received high attention by the scientific community due to their unique properties. Ceratamines are structurally relatively simple molecules and as the only substances from tubulin-binding family, they are not chiral. Given by that simple structure, they are able to cross the blood-brain barrier and act directly in the brain, which could be utilized, for example, in the treatment of Alzheimer’s disease. At present, syntheses of ceratamine analogues have been performed in order to develop novel substances with improved antiproliferative properties [159].

That is why, for example, Nodwell et al. (2010) prepared a derivative of compound **15a** with methyl groups instead of bromine atoms (compound **15c**, Figure 16). In MCF-7 cells, the IC_50_ value of compound **15c** was determined as 3 µM [161]. One of the most recent studies in this field is a study of Tao et al. (2017), who confirmed the importance of the methyl group at the C-14 and C-16 position. Derivative **15d** showed relatively good anticancer activity with the IC_50_ = 23 µM (IC_50_ of **15a** was equal to 26.5 µM) in cells from lung carcinoma (A549 cell line) [159]. A much more significant increase in the antiproliferative activity was observed for derivative **15e** (IC_50_ in A549 cells of 8.56 µM) with a benzyl group at the N-7 and substitution with three methoxy groups on the benzene ring [160].

**Figure 17:**
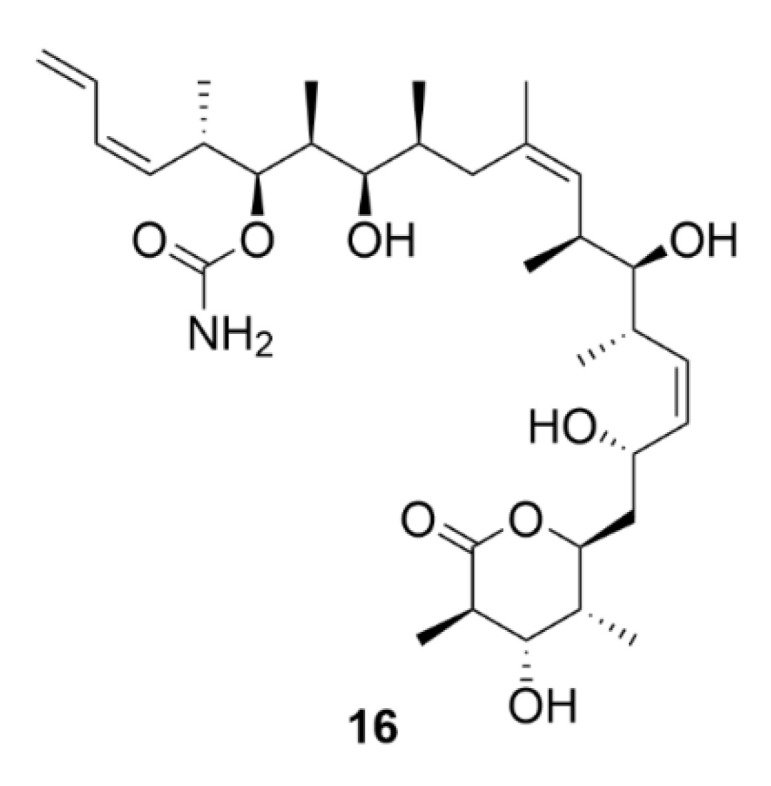
Chemical structure of discodermolide.

Another microtubule-stabilizing agent, discodermolide (compound **16**, Figure 17), originates from the marine environment. This polyhydroxylated alkatetraene lactone is a product of the marine sponge *Discodermia dissoluta*. Compound **16** is a competitive inhibitor of compound **9** acting by the same mechanism that is by microtubule polymerization induction. 

The advantage is that it also has an inhibitory effect on paclitaxel-resistant cells. In colon carcinoma cells (SW620AD-300 cell line), which are resistant to compound **9**, compound **16** is four times more toxic than that of **9** (IC_50_ = 70 nM, compound **9**—IC_50_ = 260 nM). Similarly, in resistant ovarian cancer cells (A2780AD cell line), compound **16** was more potent than compound **9** with IC_50_ = 580 nM and 3.9 µM, respectively [162]. Given the potential use of compound **16** in treatment of Taxol-resistant tumors, compound **16** has become a very intensively studied substance, which could be proven by the high number of reported syntheses [163]. However, so far, it has not found any application in clinical practice.

**Figure 18:**
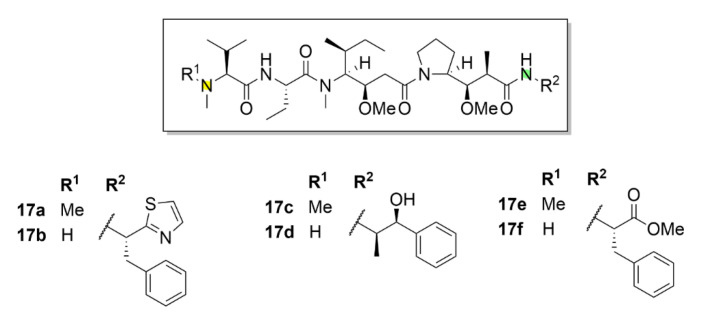
Chemical structure of dolastatin 10 and its derivatives, adapted from [173].

Dolastatin 10 (compound **17a**, Figure 18) has entered clinical trials. This short peptide was isolated and characterized as early as in 1987 [164]. Soon, its unique inhibitory properties were discovered. This compound, which is isolated from the sea hare *Dolabella auricularia*, binds to a similar binding site on tubulin as vinca alkaloids. After its discovery, its IC_50_ for mouse leukemia cells (L1210 cell line) was soon determined to be 0.5 nM, whereas for compound **14a** it was 20 nM. In addition to the ability to inhibit microtubule polymerization, the inhibitory effect of compound **17a** on tubulin-dependent guanosine triphosphate (GTP) hydrolysis was also described [165]. As aforementioned, compound **17a** soon reached clinical trials because of its high inhibitory potential. Unfortunately, its poor therapeutic index, given the relatively excessive systemic toxicity, did not allow its advancement into the third phase of clinical trials [166,167,168,169,170,171,172].

**Figure 19:**
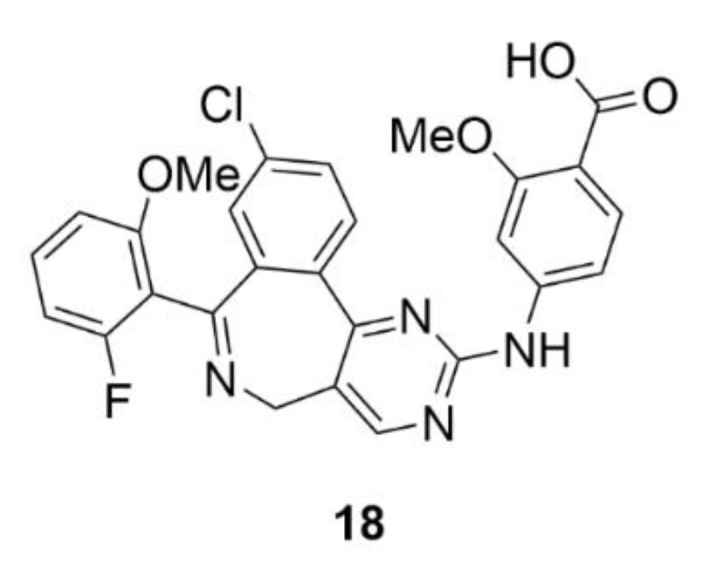
Chemical structure of alisertib.

Even despite the unsuccessful clinical trials, the research of this molecule has not stopped. On the contrary, a lot of promising derivatives of compound **17a** have been synthesized till now (Figure 19). These derivatives, named auristatins, have massively entered clinical trials. The basic auristatins were formed by modifications of the terminal amino acids in the structure of compound **17a**. It is monomethyl auristatin D (MMAD, compound **17b**, Figure 18), which differs from the parental compound **17a** by the absence of one methyl group in the *N*-terminal amino acid, auristatin E (compound **17c**, Figure 18) with norephedrine at the C-terminus, monomethyl auristatin E (MMAE, compound **17d**, Figure 18), auristatin PHE (compound **17e**, Figure 18) with phenylalanine at the C-terminus and MMAF (monomethyl auristatin PHE, compound **17f**, Figure 18) [173]. A great success in clinical practice was registered for antibody conjugates of auristatins. Doronina et al. (2003) described the need for optimization of linkers connecting the drug with the antibody targeting cancer cells, mainly given the stability of the resulting conjugate. The best results were reported for the conjugates with dipeptide linkers. A conjugate of compound **17d** with a monoclonal antibody (mAb) connected via a valine-citrulline dipeptide had sixty times higher therapeutic index than other conjugates of similar type [174]. In clinical trials, a conjugate of vedotin (compound **17d** + linker) and Brentuximab (a chimeric monoclonal antibody against the CD30 antigen) proceeded to the latest phase. This conjugate aimed for treatment of the Hodgkin lymphoma and the systemic anaplastic large-cell lymphoma is already available on the market commercialized under the trade name Adcetris^®^ [175]. The current trend is a combination of this drug with other drugs in order to reach a synergistic effect and a better therapeutic index. The example of this approach is a combination with an alkylation agent bendamustine (Treanda) in induction therapy prior to autologous stem cell transplantation in patients with Hodgkin’s lymphoma [176].

## 3. Aurora Kinases Inhibitors

Aurora kinases (AKs) belong to the group of serine/threonine kinases. So far, only three human types of these kinases have been identified: aurora kinases A, B and C. Their physiological function consists primarily in controlling cell division between the spindle formation to chromosomes division [177]. Aurora kinase A (AK-A) is located on centrosomes and on the poles of the dividing spindle. AK-A is involved in the correct progression of the cell division by many mechanisms, for example, it regulates centrosomes maturation, their identity and consequent microtubule polymerization, see [178,179]. Aurora kinase B (AK-B) is part of a larger protein complex, chromosomal passenger complex (CPC), which binds to the inner centromere space during the cell division. The function of AK-B is to control the correct distribution of sister chromatids, for example, it is involved in the stabilization of kinetochores or in the microtubule binding to kinetochore [180]. The function of aurora kinase C (AK-C) has not been fully discovered, yet. Recently, it has been shown that it plays a role mainly in the early stages of the embryonic evolution. AK-C is also incorporated in the CPC in the same way as AK-B. As AK-B is almost absent in mice and human oocytes, it is believed that AK-C plays a role in meiotic division [181]. What is common for all types of AKs is their overexpression in cancer cells, which results in cellular malfunction [177]. In addition, it has been found that AK-A phosphorylates the tumor suppressor protein p53 and, thereby, prevents its binding to the DNA and the regulation of transcription of damaged genes [182]. These findings have led to the synthesis of AK inhibitors, which could be used in cancer treatment. There are currently dozens of such low molecular weight inhibitors. Only those, for which the third phase of clinical trials have been completed or are ongoing, were selected here. For the review of AK inhibitors, see [183]. 

### 3.1. Alisertib

Alisertib (MLN8237, compound **18**, Figure 19) is one of the selective inhibitors of AK-A. The base of its structure is pyrimido-benzazepine, which induces apoptosis and induces the cell cycle arrest in the G2/M phase and in micromolar concentrations, it inhibits the AK-A activity. Compound **18** consists of an *o*-methoxybenzoic acid bound to pyrimido-benzazepine, which ensures a higher affinity to AK-A and also a greater inhibition effect in comparison to pyrimido-benzazepine alone [184]. Similarly to the parental structure, compound **18** causes the cell cycle arrest in the G2/M phase and at the same time it induces apoptosis and autophagy in many different cancer cell lines [185,186,187,188,189,190]. This mechanism of action is provided by inhibition of the synthetic pathways of mitogen-activated protein kinase (p38 MAPK), protein kinase B (Akt) and mammalian target of rapamycin (mTOR) [185]. The other mechanism of the anticancer action of compound **18** is its impact on the oncogene protein *N*-myc. It was found that AK-A stabilizes *N*-Myc by binding to it and protecting it from ubiquitination. As compound **18** changes the structure of AK-A, this stabilization does not occur and this oncogene is less expressed [191]. This could be used for example for treatment of non-small cell lung carcinoma, neuroblastoma or neurocrine prostate cancer [192]. An indisputable advantage of compound **18** is its ability to bind human serum albumin, which has a significant impact on the proper distribution of this substance in the human body [193].

Compound **18** has advanced to the latest phase of the clinical trials in the treatment of aggressive peripheral T-lymphocyte malignancies, namely, into the third phase. Its activity was compared with other commonly used chemotherapeutics for this disease, such as gemcitabine, pralatrexate, and romidepsin having distinct mechanism of action. It was shown that compound **18** is not significantly more effective than the currently used drugs [194]. At present, the second phase of clinical trials of compound **18** in patients with pleural mesothelioma (NCT02293005) is in progress, as is the second phase study comparing the activity of compound **18** co-administered with compound **9** with the activity of compound **9** alone in metastatic and recurrent breast cancer (NCT02187991). Besides tests with compound **9** co-administration, compound **18** was evaluated also with many other drugs, such as, for example, with pazopanib for the treatment of advanced solid tumors. Pazopanib is a selective inhibitor of the vascular endothelial growth factor receptor (VEGFR). The first phase of clinical trials was successfully completed with the finding of the safety and early antitumor effect of such drug combination [195]. Another drug combination of compound **18** was made with commonly used chemotherapeutics irinotecan and temozolomide for neuroblastoma treatment, which already passed the second phase of clinical trials (NCT01601535). 

In addition to confirmation of its antitumor activity, it was found that compound **18** can be administered orally in a form of a liquid solution, which could be advantageous in the treatment of pediatric patients or of patients, who have difficulties swallowing pills in general [196]. Moreover, interestingly, compound **18** could find its application in the oncolytic viral therapy, since it increases its efficacy. For example, in cancer treatment with oncolytic herpes virus, the overall antitumor activity is significantly increased when combined with compound **18**. On the one hand, it is due to the fact that viruses increase cancer cell sensitivity to compound **18**, on the other hand, compound **18** enhances the production of viruses and reduces their clearance from tumors. At the same time, compound **18** decreases the accumulation of intratumoral myeloid-derived suppressor cells, which are normally protumorigenic [197]. The efficacy of compound **18** in viral anticancer therapy has also been confirmed by using the oncolytic measles virus [198].

### 3.2. Barasertib

**Figure 20:**
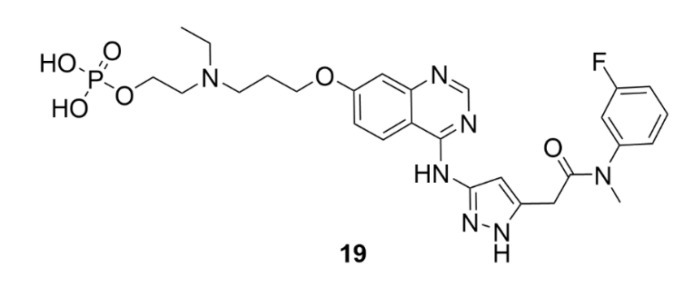
Chemical structure of barasertib.

An example of a selective inhibitor of AK-B is barasertib (AZD1152, compound **19**, Figure 20). This compound has been synthesized in 2007 by AstraZeneca and belongs to the pyrazolo quinazoline family of compounds. It is a dihydrogen phosphate prodrug, which is in plasma readily transformed into an active compound AZD1152-HQPA (AZD1152-hydroxyquinazoline pyrazol anilide) [199]. Soon after the synthesis of compound **19**, high affinity to AK-B was identified together with its ability to inhibit tumor growth. Unlike, for example, microtubule inhibitors, which arrest the cell cycle in mitosis, compound **19** induces directly apoptosis. The inhibition of AK-B causes a decreased histone H3 phosphorylation, which results in accumulation of tetraploid and polyploid cells at the tumor site and subsequent induction of apoptosis [200] The inhibition of AK-B is also associated with ROS production, which could be one of the cytotoxic mechanisms of compound **19** [201].

Recently, it was found that small miRNAs could modulate the antitumor mechanism of action of compound **19**. As aforementioned, the inhibition of AK-B leads to changes in the number of chromosomes, which could also lead to changes in miRNA expression. Some miRNAs are associated with cancer growth, while others are tumor suppressors. Zekri et al. (2018) found that cells from neuroblastoma treated with compound **19** increased tumor suppressor miRNA production, whereas they decreased production of oncogenic miRNAs [202]. However, compound **19** has not found any use in clinical practice, yet. The most advanced phase of clinical trials which compound **19** reached was the third phase (NCT00952588), for the treatment of acute myeloid leukemia (AML). Unfortunately, the study resulted in severe side effects, such as febrile neutropenia, stomatitis and pneumonia. What could increase the therapeutic index of compound **19** though is the use of nanoparticles which are being currently considered for its administration [203]. A potential positive output should be confirmed by clinical trials which are currently under preparation (NCT03217838).

## 4. Polo-like Kinases Inhibitors

Polo-like kinases (PLKs) are another group of serine/threonine kinases. The human proteome includes five types of these kinases, PLK1–PLK5. All these enzymes contain at least one common domain, the so-called polo-box. This domain is designated for targeting the enzyme to the site of its action. All PLKs are involved in cell division and control a lot of mitotic processes from the very initiation of mitosis to cytokinesis [204]. The most studied enzymes of this group are PLK4 and PLK1, the second one is also the most conserved PLK in the animal kingdom. PLK1 is expressed during the G2/M phase of the cell cycle as a result of p53-dependent transcription. It occurs after an increase in the cycline B and cycline-dependent kinase 1 levels, which, together with PLK1, ensure the proper progress of mitosis. PLK1 is involved in centrosome maturation, spindle formation, APC complex inactivation or cytokinesis regulation [205]. PLK1 includes two polo-boxes, which are responsible for its localization on centrosomes, kinetochores and the cytokinetic midbody [206]. This enzyme interacts with a protein, which binds to tumor suppressor protein p53. By phosphorylation of this binding protein, it mediates ubiquitinylation of the tumor suppressor protein p53 and thus initiates its degradation, which is one of the possible mechanisms of tumor formation [207]. By PLK1 or PLK4 inhibition, such tumorigenesis could be prevented. The use of PLK inhibitors has been considered mainly in the case of blood cell malignancies, because in leukemia, for example, cells often overexpress these enzymes [208].

PLK 2, 3 and 5 are mainly expressed in the interphase of the cell cycle and have different functions. PLK2 plays a key role in the signal transduction, synaptic plasticity, centriole duplication and cell transition from the G1 to the S phase of the cell cycle [206]; while PLK3 is involved in the cell cycle regulation, disassembly of the Golgi apparatus and in cellular stress responses [209]. PLK4 is structurally the most distinct from all other PLKs and it is a very important enzyme controlling centriole division during cell duplication [210]. The last one, PLK5, is an exceptional enzyme in the point of view that it lacks the kinase domain. However, it has the polo-box and that is why it belongs to the PLK family. It controls neuronal differentiation and also plays a crucial role in the cell cycle progression [206]. Summarized, whereas PLK2 and PLK3 are considered to be tumor suppressors, PLK1 and PLK4 are associated with carcinogenesis and are often overexpressed in tumor cells [208].

### 4.1. Volasertib

**Figure 21:**
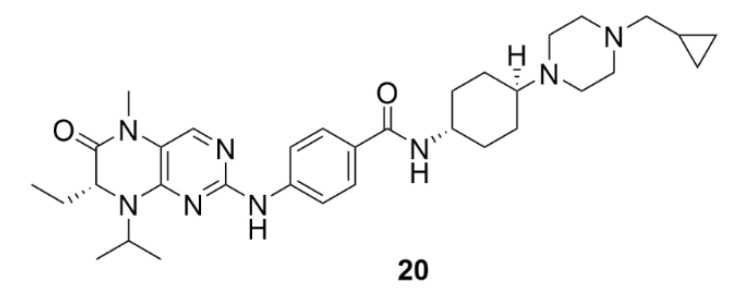
Chemical structure of volasertib.

Most inhibitors of PLKs are selectively targeted to PLK1. One of such inhibitors is Volasertib (compound **20**, Figure 21) which is currently the most clinically tested PLK inhibitor. This dihydropteridinone derivative inhibits also the activity of PLK2 and PLK3, however, at orders of magnitudes higher concentrations. Compound **20** belongs to competitive inhibitors, using the same PLK1 binding site as adenosine triphosphate [211]. Compound **20** arrests cancer cell growth in the G2/M phase of the cell cycle and induces apoptosis (NCI-H460 cell line) [212]. In clinical trials, compound **20** has been shown to be more potent in inhibiting blood cancer cells than solid tumors, for which the efficacy was very low. This difference from preclinical tests can be affected by the p53 levels. It was found that in p53-deficient cells, apoptosis is not triggered to the same extent as in case of cells with functional p53. On the contrary, in p53-deficient cells, only cell cycle arrest occurs in mitosis. In general, far greater inhibition activity of compound **20** was observed in cells with functional p53 protein [213]. Thus, compound **20** has entered the third phase of clinical trials only for the treatment of AML; this evaluation is still ongoing (NCT01721876). It was found in earlier clinical trials that pharmacokinetics of compound **20** is well predictable. Compound **20** has a very high volume of distribution and, therefore, a good ability to penetrate deep into tissues. In comparison to other drugs, it also has a longer biological half-life, which, together with good tissue distribution, leads to consideration of the possible use of compound **20** in clinical practice [214].

### 4.2. Rigosertib

**Figure 22:**
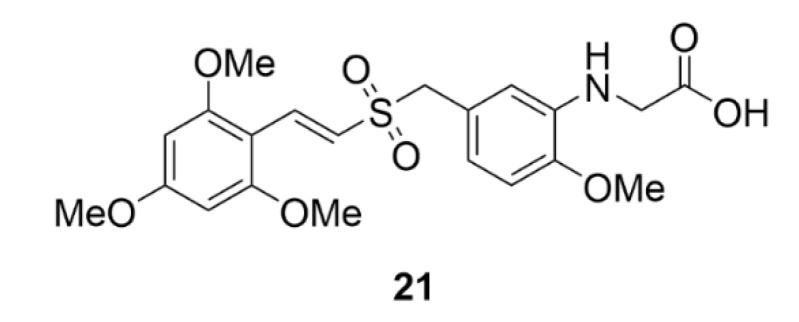
Chemical structure of Rigosertib.

Rigosertib (compound **21**, Figure 22) is an anticancer drug, the mechanism of action of which appears to be very complicated. Originally, it was identified by a screening method as a PLK1 inhibitor and till now it is incorrectly included in this group [215]. Since then, Steegmeier et al. (2017) refuted its inhibitory effect on PLK1 [216]. It was later found that this styryl benzyl sulfone substance, instead of inhibiting PLK1, interferes with signaling pathways of phosphatidylinositol 3-kinase (PI3K), which are closely related to oncogenesis [217,218]. Compound **21** also indirectly interferes with the Ras protein signaling pathway. Ras protein interacts with a conserved domain of other proteins (Ras-binding domain) and, thus, it can induce tumor progression. One of these Ras-effector proteins is also PI3K [219,220]. By using CRISPR, Jost et al. (2017) identified tubulin as a molecular target of compound **21** discovering the binding site for compound **21** on tubulin and designating it as a microtubule destabilizing agent. For these experiments, they used a commercially available compound **21** [221]. Baker et al. (2019) disproved again their statements. They found that the experiment of Jost et al. (2017) cannot be repeated with clinically used compound **21**. The purity of both compounds was then compared, and it was found that the commercially available compound **21** includes 5% of compound ON01500, which is a potent microtubule inhibitor [222]. The most probable mechanism of action and the anticancer activity of this compound though still lies in the effect on the Ras and PI3K signaling pathways.

The effects of compound **21** have been, and are being, studied in clinical trials, especially in the treatment of myelodysplastic syndrome (MDS). The commonly used treatments of MDS are hypomethylating agents, azacitidine or decitabine. Nevertheless, they are not effective for some patients; in these patients compound **21** could be a valid solution. However, in the third phase of clinical trials, it did not reduce the mortality of these patients in any way compared to the best supportive care [223]. Nowadays, further clinical testing of compound **21** is in progress (NCT02562443). Compound **21** could be potentially used in the treatment of MDS with excess blasts. In the first and second phase of clinical trials, it has been shown to promote bone marrow formation which could contribute to a reduced risk of AML disease progression. The third phase of clinical trials is now ongoing in patients responding or not responding to the treatment with hypomethylating agents (NCT01241500 and NCT01928537). A second phase of clinical trials for the combined effect of compound **21** and azacitidine in the treatment of MDS is also ongoing (NCT01926587). Besides MDS, compound **21** also finds its use in the treatment of a very aggressive head and neck squamous cell carcinoma. In preclinical in vitro studies, it was found that by affecting the PI3K pathway, compound **21** causes oxidative stress in cancer cells, induces ROS production and activates various extracellular kinases leading to cytotoxicity. At the same time, intracellular translocation of the activation transcription factor 2 was observed, which probably causes a greater sensitivity to the standard cisplatin treatment. That is why a combination therapy has been considered [224]. The first phase of clinical trials correlated with the preclinical model [225]. The results of the second phase of clinical trial have not been reported, yet (NCT01807546).

## 5. Kinesins Inhibitors

Among the proteins affecting the progression of mitosis are also molecular motors, kinesins. Their main task is to transport different molecules alongside the microtubules in a cell. Most kinesins move from the minus end of the microtubule fiber to the plus end. These movements are ensured by the conformational changes of the 15 amino acids long part, which is called the neck-linker. These conformational changes take place after cleavage of the phosphate group from the bound ATP [226]. However, there are also kinesins that transport cargo in a direction to the microtubule minus end, mainly kinesin-14 family [227]. All kinesins contain in their structure a highly conserved ATP-hydrolyzing domain and then a more diverse domain binding a transported substrate and cellular structures. There are also several kinesins that, besides transporting molecules, can control microtubule dynamics and, therefore, the whole mitosis process. We distinguish polymerizing kinesins, such as kinesin-5, and depolymerizing kinesins, such as the kinesin-13 family [228,229]. Since, in addition to their transport function, some of the kinesins also affect the mitotic spindle apparatus, they are being considered to be valid targets of anticancer drugs. There are mainly two proteins of that group, the inhibitors of which are examined in the clinical trials, namely the aforementioned kinesin-5 (eg5, KIF11) and kinesin-8, which is better known as CENP-E (centromere-associated protein E). Kinesin-5 is a homotetramer protein with the motor domains at both ends. Therefore, it is able to bind two microtubule fibers and keep them apart from each other. In the preclinical studies it was confirmed that this protein is entirely essential for cell survival. CENP-E is then important for the cell transition from the metaphase to anaphase. It plays a role in the binding of the microtubule to the kinetochore and is, therefore, essential for the correct association and alignment of chromosomes at the equatorial position [230].

### 5.1. Kinesin-5 Inhibitors

**Figure 23:**
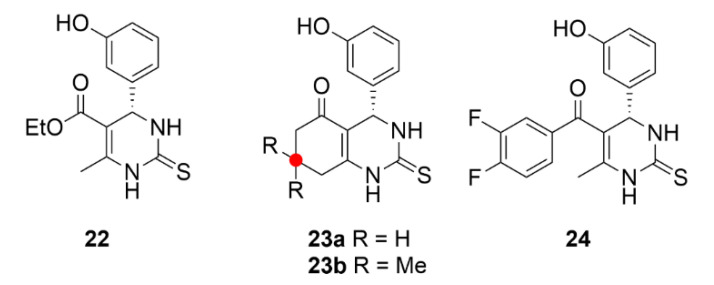
Chemical structures of kinesin-5 inhibitors.

Kinesin-5 inhibitors belong to several different groups of substances, e.g., 1,2,4-triazolones, dihydropyroles and dihydropyrazoles, dihydrothiadiazoles and dihydrooxadiazoles and many other diverse substances; for details, see [231]. The first selective inhibitor of kinesin-5 described was a dihydropyrimidine derivative monastrol (compound **22**, Figure 23). Its ability to arrest the cell cycle in mitosis was described already in 1999 [232]. However, it was found that its antimitotic activity in cancer cells is not very high, which led to a development of sundry derivatives of this compound [233]. From the dihydropyrimidine derivatives, the most potent inhibitors of kinesin-5 are enastron, dimethylenastron and fluorastrol (compounds **23a**, **23b** and **24**, respectively; Figure 23). Their enhanced potency is given mainly by increased binding specificity for kinesin-5. These derivatives differ from compound **22** mainly in the position of the original acetyl. The first two compounds mentioned are actually not substituted in this position, since instead of the acetyl group, there is a hydrogen atom, compound **24** has a difluorobenzoyl instead of the acetyl group and is the most cytotoxic one of all these derivatives (IC_50_ in HCT116 colon cancer cell line was equal to 300 nM, whereas compound **22** has the IC_50_ = 24 µM) [234]. These derivatives can be further modified, e.g., Abnous et al. (2013) synthesized a series of derivatives of dimethylenastron, which had benzylimidazolyl instead of the 3-hydroxyphenyl. The most potent derivative of these, three times as potent as dihydroenastron, has two chlorine atoms on the benzene ring and oxygen atom which replaced the original sulphur atom (IC_50_ in HeLa cells equal to 98 µM) [233].

**Figure 24:**
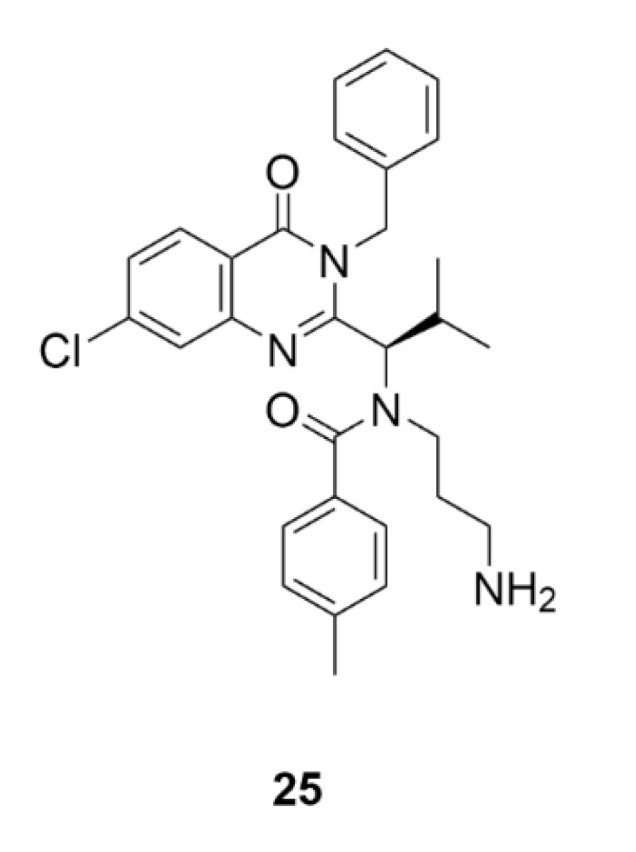
Chemical structure of ispinesib.

One of the ways to improve the cytotoxic properties of the parental compound is linking it to a fatty acid. This approach has long been applied in drug development. Such derivatives of compound **22** appeared to be in some cases (linkage to palmitic or stearic acids) up to thirteen times more effective than the unmodified compound. This improved efficacy is likely caused by a better ability to pass through cell membranes owing to the presence of lipophilic fatty acid chains [235]. The most promising group from the kinesin-5 inhibitors is quinazolines. The very first kinesin-5 inhibitor to be tested in clinical trials belongs to this group, namely, Ispinesib (compound **25**, Figure 24). Compound **25** interacts both with the microtubule-binding domain and the ATPase domain. The result of this binding is weakening of the kinesin interaction with microtubules and arresting the motor activity in ADP-bound state. Compound **25** has passed through sixteen clinical trials till now, fifteen of which have been completed. So far, in none of the cases, compound **25** has moved further than to the second phase of clinical trials because its effects in clinical practice are not convincing [236,237,238]. However, a lot of other small molecules inhibiting kinesin-5 reached clinical trials, such as, AZD4877, Filanesib, SB-743921, Litronesib, MK-0731 or EMD 534085. Nevertheless, none of these substances have progressed beyond the second phase of the clinical trials [239,240,241,242,243,244].

### 5.2. CENP-E Inhibitors

**Figure 25:**
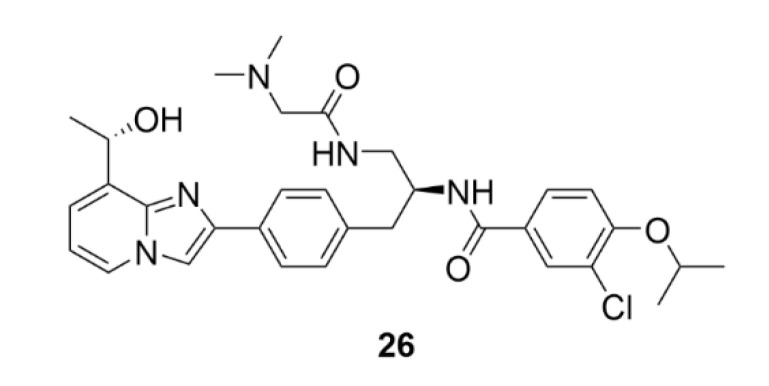
Chemical structure of GSK923295.

Even though inhibitors of kinesin-8, or CENP-E, are not as studied as the aforementioned inhibitors of kinesin-5, it is an interesting family of compounds. However, till now, only one compound has entered clinical trial, namely an allosteric inhibitor of the motor domain of CENP-E coded GSK923295 (compound **26**, Figure 25). This molecule inhibits the release of a phosphate from ATP and, thus, it stabilizes the interaction of the motor domain with microtubules. This causes the inability to align chromosomes at the equatorial plane in metaphase and, thus, arrests cell mitosis. The inhibition effect of this compound has been evaluated in more than 200 cell lines, so far, with the GI_50_ (concentration of a compound causing 50% inhibition of cell growth) in nanomolar ranges; for details, see supplementary information in [245]. In the first clinical study in patients with previously treated solid tumors, any severe side effects were not confirmed, therefore, further clinical testing has been proposed [246].

**Figure 26:**
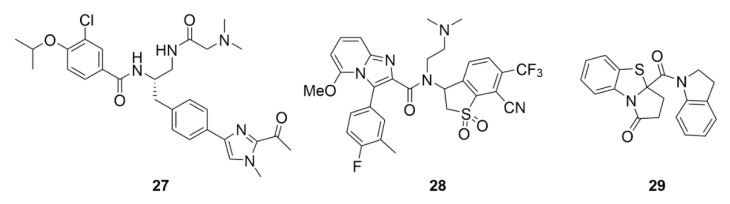
Chemical structure of PF-2771, Compound A and benzo[*d*]pyrrolo [2,1-*b*]thiazole derivative, adapted from [247,248].

To the group of the CENP-E inhibitors belongs also PF-2771 (compound **27**, Figure 26). This compound has been tested to find out whether inhibition of CENP-E is a proper therapeutic approach for treatment of triple-negative or basal breast cancer, the cells of which overexpress this kinesin. Compound **27** was shown to be a very selective and effective CENP-E inhibitor. It is effective against cells from the panel of breast cancer cell lines (EC_50_ max. 10 µM), and also against mouse models with HCC1806 xenografts [247]. Compound **27** has never reached clinical trials. Ohashi et al. (2015) reported the biological activity of a new CENP-E inhibitor synthesized in Takeda Pharmaceutical Company Ltd. named simply Compound-A (compound **28**, Figure 26). Compound **28** inhibited the activity of the ATPase motor domain and caused chromosome misalignment at the equatorial plane and cell cycle arrest in mitosis. The antiproliferative activity of compound **28** was confirmed in 18 different cancer cell lines and also in mouse models [248]. The most recent work in the field reported on CENP-E inhibitors is the study of Yamane et al. (2019), who identified the benzo[*d*]pyrrolo [2,1-*b*]thiazole derivative (compound **29**, Figure 26), which acts by a similar mechanism as the aforementioned compounds and, at the same time, it was found that it induces apoptosis. The antiproliferative activity of compound **29** was determined in HeLa and HCT116 cells with IC_50_ of 44 and 90 µM, respectively, whereas in non-cancerous cells (WI-38), the IC_50_ was significantly higher, of 200 µM, thus, the compound exhibits cancer cell selectivity [249].

## 6. Summary and Outlook

Enormous progress has been made since the discovery and isolation of the first antimitotic drugs, such as compound **1**, and identification of their mechanism of action. In the recent decade, it has been compound **9** which has been designated as the leading drug for treatment of various cancer diseases. However, due to its limited source, poor solubility and excessive side toxicity, novel derivatives are being developed and evaluated. In addition, there is a continuous search for novel compounds with a similar mechanism of action as well as repurposing of “old” drugs, such as colchicine, now being administered for different indications. In this review article, compounds being designated as mitotic poisons were described not only regarding their chemical structure, mechanism of action, and clinical relevance, but also in terms of the development of their derivatives with improved properties. Mitotic poisons, similar to, e.g., sarco/endoplasmic reticulum Ca^2+^ ATPase (SERCA) inhibitors [250,251], are compounds of high importance in cancer research and treatment, and therefore, it is obvious that novel synthetic and semi-synthetic approaches for higher production of the active or fine-tuned ones will emerge [252]. Some of these will hopefully be both cancer cell selective and clinically safe and, thus, could offer a new hope for the treatment of, so far, untreatable diseases in terms of cancer or at least they could offer a better disease outcome.

## Figures and Tables

**Table 1:** Number of clinical trials per compound mentioned irrespective of condition.

Compound	Number of Clinical Trials ^1^
Colchicine	171
Paclitaxel	3882
Docetaxel	2481
Epothilones	112
Vinblastine	208
Vincristine	1150
Alisertib	60
Barasertib (AZD1152)	10
Rigosertib	38
Ispinesib	16

^1^ till July 2020.

**Table 2:** Selected clinical trials on mitotic inhibitors [14].

Compound	Condition	Status	Phase	Clinical Trial Identifier
**Colchicine**	Gout	Completed	IV	NCT01451645 NCT02060552 NCT01112982 NCT02500641 NCT02995512 NCT01310673
	Familiar Mediterranean fever			NCT02602028
	Hepatocellular carcinoma	Recruiting	II	NCT04264260
**Paclitaxel**	Non-small cell lung cancer	Completed	IV	NCT02151149 NCT00686322 NCT03092986
	Carcinoma, large cell Neuroendocrine tumors			NCT01317615
	Breast cancer			NCT01094184 NCT01301729
	Cancer			NCT00606515
**Docetaxel**	Non-small cell lung cancer	Completed	IV	NCT01442909 NCT00883675
	Breast cancer			NCT02502864 NCT02445586 NCT01660542 NCT01094184 NCT01301729
	Prostatic neoplasm			NCT00280098
	Head and neck neoplasm			NCT00772681
	Squamous cell carcinoma			NCT02088515
**Ixabepilone**	Breast cancer	Completed	III	NCT00082433 NCT00080301
				NCT00789581
**Vinblastine**	Non-small cell lung cancer	Completed	IV	NCT03092986
**Vincristine**	Acute lymphoblastic leukemia	Completed	IV	NCT00411541 NCT00526305 NCT00526175 NCT00199069 NCT00526409 NCT00494897 NCT00853008 NCT02036489
	Acute lymphocytic leukemia			NCT00199069 NCT00199004 NCT00199095 NCT00199056
	Immune thrombocytopenia			NCT03229746
	Lymphoma			NCT00199017
**Vedotin/** **Brentuximab**	Hodgkin lymphoma	Completed	IV	NCT01990534
		Active	III	NCT01712490
	Anaplastic large-cell lymphoma	Recruiting	IV	NCT01909934
		Active	III	NCT01777152
		Completed		NCT01578499
**Alisertib**	Peripheral T-cell lymphoma	Completed	III	NCT01482962
**Barasertib**	Acute myeloid leukemia	Completed	III	NCT00952588
**Volasertib**	Acute myeloid leukemia	Active	III	NCT01721876
**Rigosertib**	Metastatic Pancreatic Adenocarcinoma	Completed	III	NCT01360853
	Myelodysplastic syndrome			NCT01928537 NCT01241500
**Ispinesib**	Renal cell cancer Breast cancer Head and neck cancer Ovarian cancer Prostate cancer Non-small cell lung cancer Melanoma (skin) Liver cancer Metastatic colorectal cancer	Completed	II	NCT00354250 NCT00089973 NCT00095628 NCT00097409 NCT00096499 NCT00085813 NCT00095953 NCT00095992 NCT00103311

**Table 3:** IC_50_ values of colchicine derivatives **2a**–**2j** (the chemical structures are depicted in Figure 4) in cancerous and noncancerous cell lines evaluated in vitro after 72 h of treatment; the data are from [26].

	Cancer Cells				Normal Cells
	A549	MCF-7	LoVo	LoVo/DX	BALB/3T3
Comp.	IC_50_ (µM)				
**2a**	0.010 ± 0.0001	0.015 ± 0.002	0.014 ± 0.004	0.135 ± 0.012	0.103 ± 0.089
**2b**	0.115 ± 0.007	0.178 ± 0.020	0.125 ± 0.044	0.700 ± 0.088	1.260 ± 0.796
**2c**	0.074 ± 0.009	0.057 ± 0.011	0.074 ± 0.019	1.010 ± 0.020	0.104 ± 0.043
**2d**	0.010 ± 0.0001	0.013 ± 0.002	0.007 ± 0.002	0.050 ± 0.010	0.066 ± 0.031
**2e**	0.012 ± 0.004	0.018 ± 0.002	0.011 ± 0.004	0.071 ± 0.010	0.102 ± 0.063
**2f**	0.030 ± 0.021	0.055 ± 0.026	0.018 ± 0.010	0.074 ± 0.007	0.138 ± 0.010
**2g**	0.012 ± 0.004	0.027 ± 0.007	0.011 ± 0.0001	0.072 ± 0.011	0.116 ± 0.009
**2h**	0.089 ± 0.020	0.132 ± 0.017	0.054 ± 0.017	0.089 ± 0.026	0.173 ± 0.108
**2i**	0.095 ± 0.005	0.125 ± 0.014	0.062 ± 0.013	0.091 ± 0.009	0.146 ± 0.014
**2j**	0.093 ± 0.014	0.125 ± 0.015	0.281 ± 0.185	4.240 ± 1.330	0.135 ± 0.015

## Abbreviations

A549: human cells from lung carcinoma; AKs, Aurora kinases; AK-A,B,C, Aurora kinase A, B, C; AML, acute myeloid leukemia; APC, anaphase-promoting complex; ATP, adenosine triphosphate; Caco-2, human cells from human epithelial colorectal adenocarcinoma; CD30, a protein of the tumor necrosis factor receptor family; CENP-E, centromere-associated protein E; CHOP, doxorubicin-based chemotherapy protocol; CNS, central nervous system; CPC, chromosomal passenger complex; CRISPR, clustered regularly interspaced short palindromic repeats; CYP, cytochrome P450; EC_50_, half maximal effective concentration; Eg5, kinesin-related motor protein; EGFR, epidermal growth factor receptor; EpoA–EpoF, epothilones A–F; GI_50_, the concentration causing 50% cell growth inhibition; GTP, guanosine triphosphate; HCT116, human cells from colon carcinoma; HEK 293T, human embryonic kidney cells; HeLa, human cells from cervical carcinoma; HT-29, human cells from colorectal carcinoma; hTERT, human telomerase reverse transcriptase; Huh7, human cells from liver; IC_50_, half maximal inhibitory concentration; IC_70_, the concentration inhibiting the growth by 70%; KIF11, kinesin family member 11; L1210, mouse cells from lymphocytic leukemia; LoVo, human cells from colon cancer; LoVo/DX, multi-drug resistant human cells from colon cancer; MAPK, mitogen-activated protein kinase; MCF-7, human cells from breast carcinoma; MDS, myelodysplastic syndrome; MiaPaCa-2, human cells from pancreas adenocarcinoma; MMAD, monomethyl auristatin D; MMAE, monomethyl auristatin E; MMAF, monomethyl auristatin F; mTOR, mammalian target of rapamycin; NCI-H1299, human cells from lung carcinoma; NCI-H460, human cells from lung carcinoma; NGS, next-generation sequencing; OV-90, human cells from ovary carcinoma; PANC-1, human cells from pancreas adenocarcinoma; PEG, polyethylene glycol; PI3K, phosphoinositide 3-kinase; PLK1-5, polo-like kinases 1-5; PTM, post-translational modifications; ROS, reactive oxygen species; SERCA, sarco/endoplasmic reticulum Ca^2+^ ATPase; SKOV-3, human cells from ovary; SW620AD-300, human cells from colon cancer; T3M4, human cells from pancreatic ductal adenocarcinoma; TALE-TF, transcription activator-like effectors-transcriptional factor; VCRT, combination of vinblastine, cisplatin and radiation therapy; VEGF, vascular endothelial growth factor; VEGFR, the vascular endothelial growth factor receptor; WHO, World Health Organization; WI-38, human lung fibroblasts.

## References

[1] WHO. https://www.who.int/news-room/fact-sheets/detail/cancer.

[2] Dumbrava E.I., Meric-Bernstam F. (2018). Personalized cancer therapy-leveraging a knowledge base for clinical decision-making. Cold Spring Harb. Mol. Case Stud..

[3] WHO. https://www.who.int/medicines/publications/essentialmedicines/en/.

[4] Cahill D.P., Lengauer C., Yu J., Riggins G.J., Willson J.K., Markowitz S.D., Kinzler K.W., Vogelstein B. (1998). Mutations of mitotic checkpoint genes in human cancers. Nature.

[5] Chen X., Widmer L.A., Stangier M.M., Steinmetz M.O., Stelling J., Barral Y. (2019). Remote control of microtubule plus-end dynamics and function from the minus-end. eLife.

[6] Li C., Li J., Goodson H.V., Alber M.S. (2014). Microtubule dynamic instability: The role of cracks between protofilaments. Soft Matter..

[7] Strothman C., Farmer V., Arpağ G., Rodgers N., Podolski M., Norris S., Ohi R., Zanic M. (2019). Microtubule minus-end stability is dictated by the tubulin off-rate. J. Cell Biol..

[8] Ferro L.S., Can S., Turner M.A., ElShenawy M.M., Yildiz A. (2019). Kinesin and dynein use distinct mechanisms to bypass obstacles. eLife.

[9] Stearns T., Evans L., Kirschner M. (1991). γ-Tubulin is a highly conserved component of the centrosome. Cell.

[10] Findeisen P., Mühlhausen S., Dempewolf S., Hertzog J., Zietlow A., Carlomagno T., Kollmar M. (2014). Six subgroups and extensive recent duplications characterize the evolution of the eukaryotic tubulin protein family. Genome Biol. Evol..

[11] Ross I., Clarissa C., Giddings T.H., Winey M. (2013). ε-tubulin is essential in *Tetrahymena thermophila* for the assembly and stability of basal bodies. J. Cell Sci..

[12] Garreau de Loubresse N., Ruiz F., Beisson J., Klotz C. (2001). Role of delta-tubulin and the C-tubule in assembly of *Paramecium* basal bodies. BMC Cell Biol..

[13] Song Y., Brady S.T. (2015). Post-translational modifications of tubulin: Pathways to functional diversity of microtubules. Trends Cell Biol..

[14] Clinical Trials. http://clinicaltrials.gov.

[15] Hartung E.F. (1954). History of the use of colchicum and related medicaments in gout; with suggestions for further research. Ann. Rheum. Dis..

[16] Graham W., Roberts J.B. (1953). Intravenous colchicine in the management of gouty arthritis. Ann. Rheum. Dis..

[17] Eigsti O.J., Dustin P., Gay-winn N. (1949). On the discovery of the action of colchicine on mitosis in 1889. Science.

[18] Spasevska I., Ayoub A.T., Winter P., Preto J., Wong G.K.-S., Dumontet C., Tuszynski J.A. (2017). Modeling the *Colchicum autumnale* tubulin and a comparison of its interaction with colchicine to human tubulin. Int. J. Mol. Sci..

[19] Bhattacharyya B., Panda D., Gupta S., Banerjee M. (2008). Anti-mitotic activity of colchicine and the structural basis for its interaction with tubulin. Med. Res. Rev..

[20] Ateş F.B., Özmen N., Sezginer E.K., Kurt E.E. (2018). Effects of colchicine on cell cycle arrest and MMP-2 mRNA expression in MCF 7 breast adenocarcinoma cells. Turk. Bull. Hyg. Exp. Biol..

[21] McCarty D.J. (2008). Urate crystals, inflammation, and colchicine. Arthritis Rheum..

[22] Paschke S., Weidner A.F., Paust T., Marti O., Beil M., Ben-Chetrit E. (2013). Technical advance: Inhibition of neutrophil chemotaxis by colchicine is modulated through viscoelastic properties of subcellular compartments. J. Leukoc. Biol..

[23] Dinarello C.A., Wolff S.M., Goldfinger S.E., Dale D.C., Alling D.W. (1974). Colchicine therapy for familial mediterranean fever. A double-blind trial. N. Engl. J. Med..

[24] Filkenstein Y., Aks S.E., Hutson J.R., Juurlink D.N., Nguyen P., Dubnov-Raz G., Pollak U., Koren G., Bentur Y. (2010). Colchicine poisoning: The dark side of an ancient drug. Clin. Toxicol..

[25] Bhattacharya S., Das A., Datta S., Ganguli A., Chakrabarti G. (2016). Colchicine induces autophagy and senescence in lung cancer cells at clinically admissible concentration: Potential use of colchicine in combination with autophagy inhibitor in cancer therapy. Tumor Biol..

[26] Majcher U., Klejborowska G., Kaik M., Maj E., Wietrzyk J., Moshari M., Preto J., Tuszynski J.A., Huczyński A. (2018). Synthesis and biological evaluation of novel triple-modified colchicine derivatives as potent tubulin-targeting anticancer agents. Cells.

[27] Kang G.J., Getahun Z., Muzaffar A., Brossi A., Hamel E. (1990). *N*-acetylcolchinol *O*-methyl ether and thiocolchicine, potent analogs of colchicine modified in the C ring. Evaluation of the mechanistic basis for their enhanced biological properties. J. Biol. Chem..

[28] Yasobu N., Kitajima M., Kogure N., Shishido Y., Matsuzaki T., Nagaoka M., Takayama H. (2011). Design, synthesis, and antitumor activity of 4-halocolchicines and their pro-drugs activated by cathepsin B. ACS Med. Chem. Lett..

[29] Larocque K., Ovadje P., Djurdjevic S., Mehdi M., Green J., Pandey S. (2014). Novel analogue of colchicine induces selective pro-death autophagy and necrosis in human cancer cells. PLoS ONE.

[30] Davis P.D., Dougherty G.J., Blakey D.C., Galbraith S.M., Tozer G.M., Holder A.L., Naylor M.A., Nolan J., Stratford M.R.L., Chaplin D.J. (2002). ZD6126, A novel vascular-targeting agent that causes selective destruction of tumor vasculature. Cancer Res..

[31] Gracheva I.A., Svirshchevskaya E.V., Faerman V.I., Beletskaya I.P., Fedorov A.Y. (2018). Synthesis and antiproliferative properties of bifunctional allocolchicine derivatives. Synthesis.

[32] Lippert J.W. (2007). Vascular disrupting agents. Bioorg. Med. Chem..

[33] Marzo-Mas A., Falomir E., Murga J., Carda M., Marco J.A. (2018). Effects on tubulin polymerization and down-regulation of c-Myc, hTERT and VEGF genes by colchicine haloacetyl and haloaroyl derivatives. Eur. J. Med. Chem..

[34] Cortese M., Goellner S., Acosta E.G., Neufeldt C.J., Oleksiuk O., Lampe M., Haselmann U., Funaya C., Schieber N., Ronchi P. (2017). Ultrastructural characterization of zika virus replication factories. Cell Rep..

[35] Richter M., Boldescu V., Graf D., Streicher F., Dimoglo A., Bartenschlager R., Klein C.D. (2019). Synthesis, biological evaluation, and molecular docking of combretastatin and colchicine derivatives and their hCE1-activated prodrugs as antiviral agents. ChemMedChem.

[36] Choi M.Y., Wee Y.M., Kim Y.H., Shin S., Yoo S.E., Han D.J. (2019). Novel colchicine derivatives enhance graft survival after transplantation via suppression of T-cell differentiation and activity. J. Cell Biochem..

[37] Van Tamelen E.E., Spencer T.A., Allen D.S., Orvis R.L. (1959). The total synthesis of colchicine. J. Am. Chem. Soc..

[38] Schreiber J., Leimgruber W., Pesaro M., Schudel P., Eschenmoser A. (1959). Synthese des colchicins. Angew. Chem..

[39] Chen B., Liu X., Hu Y.J., Zhang D.M., Deng L., Lu J., Min L., Ye W.C., Li C.C. (2017). Enantioselective total synthesis of (−)-colchicine, (+)-demecolcinone and metacolchicine: Determination of the absolute configurations of the latter two alkaloids. Chem. Sci..

[40] Pandey D.K., Banik R.M. (2012). Optimization of extraction conditions for colchicine from *Gloriosa superba* tubers using response surface methodology. J. Agric. Technol..

[41] Kefi S. (2018). A novel approach for production of colchicine as a plant secondary metabolite by in vitro plant cell and tissue cultures. J. Agric. Sci. Technol. A.

[42] Sivakumar G., Alba K., Phillips G.C. (2017). Biorhizome: A biosynthetic platform for colchicine biomanufacturing. Front. Plant Sci..

[43] Wani M.C., Taylor H.L., Wall M.E., Coggon P., McPhail A.T. (1971). Plant antitumor agents. VI. Isolation and structure of taxol, a novel antileukemic and antitumor agent from *Taxus brevifolia*. J. Am. Chem. Soc..

[44] Parness J., Horwitz S.B. (1981). Taxol binds to polymerized tubulin in vitro. J. Cell Biol..

[45] Jordan M.A., Toso R.J., Thrower D., Wilson L. (1993). Mechanism of mitotic block and inhibition of cell-proliferation by taxol at low concentrations. Proc. Natl. Acad. Sci. USA.

[46] Ozols R.F., Bundy B.N., Greer B.E., Fowler J.M., Clarke-Pearson D., Burger R.A., Mannel R.S., DeGeest K., Hartenbach E.M., Baergen R. (2003). Phase III trial of carboplatin and paclitaxel compared with cisplatin and paclitaxel in patients with optimally resected stage III ovarian cancer: A gynecologic oncology group study. J. Clin. Oncol..

[47] Weiss R.B., Donehower R.C., Wiernik P.H., Ohnuma T., Gralla R.J., Trump D.L., Baker J.R., Van Echo D.A., Von Hoff D.D., Leyland-Jones B. (1990). Hypersensitivity reactions from taxol. J. Clin. Oncol..

[48] Miele E., Spinelli G.P., Miele E., Tomao F., Tomao S. (2009). Albumin-bound formulation of paclitaxel (Abraxane^®^ ABI-007) in the treatment of breast cancer. Int. J. Nanomedicine..

[49] Du X., Yin S., Xu L., Ma J., Yu H., Wang G., Li J. (2020). Polylysine and cysteine functionalized chitosan nanoparticle as an efficient platform for oral delivery of paclitaxel. Carbohydr. Polym..

[50] Zhao M., Li H., Fan L., Ma Y., Gong H., Lai W., Fang Q., Hu Z. (2018). Quantitative proteomic analysis to the first commercialized liposomal paclitaxel nano-platform Lipusu revealed the molecular mechanism of the enhanced anti-tumor effect. Artif. Cells Nanomed. Biotechnol..

[51] Ranade A.A., Joshi D.A., Phadke G.K., Patil P.P., Kasbekar R.B., Apte T.G., Dasare R.R., Mengde S.D., Parikh P.M., Bhattacharyya G.S. (2013). Clinical and economic implications of the use of nanoparticle paclitaxel (Nanoxel) in India. Ann. Oncol..

[52] Galletti E., Magnani M., Renzulli M.L., Botta M. (2007). Paclitaxel and docetaxel resistance: Molecular mechanisms and development of new generation taxanes. ChemMedChem.

[53] Clarke S.J., Rivory L.P. (1999). Clinical pharmacokinetics of docetaxel. Clin. Pharmacokinet..

[54] Valero V., Jones S.E., Von Hoff D.D., Booser D.J., Mennel R.G., Ravdin P.M., Holmes F.A., Rahman Z., Schottstaedt M.W., Erban J.K. (1998). A phase II study of docetaxel in patients with paclitaxel-resistant metastatic breast cancer. J. Clin. Oncol..

[55] Leonelli F., La Bella A., Migneco L.M., Bettolo R.M. (2008). Design, synthesis and applications of hyaluronic acid-paclitaxel bioconjugates. Molecules.

[56] Safavy A., Raisch K.P., Khazaeli M.B., Buchsbaum D.J., Bonner J.A. (1999). Paclitaxel derivatives for targeted therapy of cancer: Toward the development of smart taxanes. J. Med. Chem..

[57] Nakamura J., Nakajima N., Matsumura K., Hyon S.H. (2010). Water-soluble taxol conjugates with dextran and targets tumor cells by folic acid immobilization. Anticancer Res..

[58] Darmostuk M., Rimpelova S., Gbelcova H., Ruml T. (2015). Current approaches in SELEX: An update to aptamer selection technology. Biotechnol. Adv..

[59] Li F., Lu J., Liu J., Liang C., Wang M., Wang L., Li D., Yao H., Zhang Q., Wen J. (2017). A water-soluble nucleolin aptamer-paclitaxel conjugate for tumor-specific targeting in ovarian cancer. Nat. Commun..

[60] Zhang C., Zhang S., Zhi D., Zhao Y., Cui S., Cui J. (2020). Co-delivery of paclitaxel and survivin siRNA with cationic liposome for lung cancer therapy. Colloid Surf. A-Physicochem. Eng. Asp..

[61] Lee J., Chae S.W., Ma L.J., Lim S.Y., Alnajjar S., Choo H.Y.P., Lee H.J., Rhie S.J. (2019). Pharmacokinetic alteration of paclitaxel by ferulic acid derivative. Pharmaceutics.

[62] Kubo T., Nogami N., Bessho A., Morita A., Ikeo S., Yokoyama T., Ishihara M., Honda T., Fujimoto N., Murakami S. (2019). Phase II trial of carboplatin, nab-paclitaxel and bevacizumab for advanced non-squamous non-small cell lung cancer (CARNAVAL study; TORG1424/OLCSG1402). Ann. Oncol..

[63] Redondo A., Colombo N., Dreosti L.M., McCormack M., Nogeira Rodrigues A., Scambia G., Roszak A., Donica M., Ulker B., González Martín A. (2019). Primary results from CECILIA, a global single-arm phase II study evaluating bevacizumab (BEV), carboplatin (C) and paclitaxel (P) for advanced cervical cancer (aCC). Ann. Oncol..

[64] González Martín A., Oza A.M., Embleton A.C., Pfisterer J., Ledermann J.A., Pujade-Lauraine E., Kristensen G., Bertrand M.A., Beale P., Cervantes A. (2019). ICON7 investigators. Exploratory outcome analyses according to stage and/or residual disease in the ICON7 trial of carboplatin and paclitaxel with or without bevacizumab for newly diagnosed ovarian cancer. Gynecol. Oncol..

[65] El-Sayed E.R., Ahmed A.S., Hassan I.A., Ismaiel A.A., El-Din A.A.K. (2019). Strain improvement and immobilization technique for enhanced production of the anticancer drug paclitaxel by *Aspergillus fumigatus* and *Alternaria tenuissima*. Appl. Microbiol. Biotechnol..

[66] Holton R.A., Somoza C., Kim H.B., Liang F., Biediger R.J., Boatman D., Shindo M., Smith C.C., Kim S., Nadizadeh H. (1994). First total synthesis of taxol. 1. Functionalization of the B ring. J. Am. Chem. Soc..

[67] Nicolaou K.C., Yang Z., Liu J.J., Ueno H., Nantermet P.G., Guy R.K., Claiborne C.F., Renaud J., Couladouros E.A., Paulvannan K. (1994). Total synthesis of taxol. Nature.

[68] Danishefsky S.J., Masters J.J., Young W.B., Link J.T., Snyder L.B., Magee T.V., Jung D.K., Isaacs R.C.A., Bornmann W.G., Alaimo C.A. (1996). Total synthesis of baccatin III and taxol. J. Am. Chem. Soc..

[69] Wender P.A., Badham N.F., Conway S.P., Floreancig P.E., Glass T.E., Gränicher C., Houze J.B., Jänichen J., Lee D., Marquess D.G. (1997). The pinene path to taxanes. 5. Stereocontrolled synthesis of a versatile taxane precursor. J. Am. Chem. Soc..

[70] Morihira K., Hara R., Kawahara S., Nishimori T., Nakamura N., Kusama H., Kuwajima I. (1998). Enantioselective total synthesis of taxol. J. Am. Chem. Soc..

[71] Isamu S., Hayato I., Hiroki S., Masatoshi H., Yu-ichirou T., Teruaki M. (1998). A new method for the synthesis of baccatin III. Chem. Lett..

[72] Doi T., Fuse S., Miyamoto S., Nakai K., Sasuga D., Takahashi T. (2006). A formal total synthesis of taxol aided by an automated synthesizer. Chem. Asian J..

[73] Fukaya K., Kodama K., Tanaka Y., Yamazaki H., Sugai T., Yamaguchi Y., Watanabe A., Oishi T., Sato T., Chida N. (2015). Synthesis of paclitaxel. 2. Construction of the ABCD ring and formal synthesis. Org. Lett..

[74] Hirai S., Utsugi M., Iwamoto M., Nakada M. (2015). Formal total synthesis of (−)-taxol through Pd-catalyzed eight-membered carbocyclic ring formation. Chem. Eur. J..

[75] Patel R.N. (1998). Tour de paclitaxel: Biocatalysis for semisynthesis. Annu. Rev. Microbiol..

[76] Ganem B., Franke R.R. (2007). Paclitaxel from primary taxanes: A perspective on creative invention in organozirconium chemistry. J. Org. Chem..

[77] Kumar P., Singh B., Thakur V., Thakur A., Thakur N., Pandey D., Chand D. (2019). Hyper-production of taxol from *Aspergillus fumigatus*, an endophytic fungus isolated from *Taxus* sp. of the Northern Himalayan region. Biotechnol. Rep..

[78] Soliman S.S.M., Raizada M.N. (2018). Darkness: A crucial factor in fungal taxol production. Front. Microbiol..

[79] Mooberry S.L., Tien G., Hernandez A.H., Plubrukarn A., Davidson B.S. (1999). Laulimalide and isolaulimalide, new paclitaxel-like microtubule stabilizing agents. Cancer Res..

[80] Churchill C.D.M., Klobukowski M., Tuszynski J.A. (2015). The unique binding mode of laulimalide to two tubulin protofilaments. Chem. Biol. Drug Des..

[81] Castro-Alvarez A., Pineda O., Vilarrasa J. (2018). Further insight into the interactions of the cytotoxic macrolides laulimalide and peloruside A with their common binding site. ACS Omega.

[82] Pryor D.E., O’Brate A., Bilcer G., Díaz J.F., Wang Y., Wang Y., Kabaki M., Jung M.K., Andreu J.M., Ghosh A.K. (2002). The microtubule stabilizing agent laulimalide does not bind in the taxoid site, kills cells resistant to paclitaxel and epothilones, and may not require its epoxide moiety for activity. Biochemistry.

[83] Clark E.A., Hills P.M., Davidson B.S., Wender P.A., Mooberry S.L. (2006). Laulimalide and synthetic laulimalide analogues are synergistic with paclitaxel and 2-methoxyestradiol. Mol. Pharm..

[84] Liu J., Towle M.J., Cheng H., Saxton P., Reardon C., Wu J., Murphy E.A., Kuznetsov G., Johannes C.W., Tremblay M.R. (2007). In vitro and in vivo anticancer activities of synthetic (−)-laulimalide, a marine natural product microtubule stabilizing agent. Anticancer Res..

[85] Paterson I., Menche D., Håkansson A.E., Longstaff A., Wong D., Barasoain I., Buey R.M., Díaz J.F. (2005). Design, synthesis and biological evaluation of novel, simplified analogues of laulimalide: Modification of the side chain. Bioorg. Med. Chem. Lett..

[86] Paterson I., Bergmann H., Menche D., Berkessel A. (2004). Synthesis of novel 11-desmethyl analogues of laulimalide by Nozaki-Kishi coupling. Org. Lett..

[87] Mooberry S.L., Randall-Hlubek D.A., Leal R.M., Hegde S.G., Hubbard R.D., Zhang L., Wender P.A. (2004). Microtubule-stabilizing agents based on designed laulimalide analogues. Proc. Natl. Acad. Sci. USA.

[88] Wender P.A., Hegde S.G., Hubbard R.D., Zhang L., Mooberry S.L. (2003). Synthesis and biological evaluation of (−)-laulimalide analogues. Org. Lett..

[89] Gallagher B.M., Fang F.G., Johannes C.W., Pesant M., Tremblay M.R., Zhao H., Akasaka K., Li X., Liu J., Littlefield B.A. (2004). Synthesis and biological evaluation of (−)-laulimalide analogues. Bioorg. Med. Chem. Lett..

[90] Ahmed A., Hoegenauer E.K., Enev V.S., Hanbauer M., Kaehlig H., Öhler E., Mulzer J. (2003). Total synthesis of the microtubule stabilizing antitumor agent laulimalide and some nonnatural analogues: The power of sharpless’ asymmetric epoxidation. J. Org. Chem..

[91] Crimmins M.T., Stanton M.G., Allwein S.P. (2002). Asymmetric total synthesis of (−)-laulimalide: Exploiting the asymmetric glycolate alkylation reaction. J. Am. Chem. Soc..

[92] Enev V.S., Kaehlig H., Mulzer J. (2001). Macrocyclization via allyl transfer: Total synthesis of laulimalide. J. Am. Chem. Soc..

[93] Ghosh A.K., Wang Y. (2000). Total synthesis of (−)-laulimalide. J. Am. Chem. Soc..

[94] Mulzer J., Hanbauer M. (2002). Total synthesis of the antitumor agent (−)-laulimalide. Tetrahedron Lett..

[95] Nelson S.G., Cheung W.S., Kassick A.J., Hilfiker M.A. (2002). A *de novo* enantioselective total synthesis of (−)-laulimalide. J. Am. Chem. Soc..

[96] Paterson I., De Savi C., Tudge M. (2001). Total synthesis of the microtubule-stabilizing agent (−)-laulimalide. Org. Lett..

[97] Trost B.M., Seganish W.M., Chung C.K., Amans D. (2012). Total synthesis of laulimalide: Synthesis of the Northern and Southern fragments. Chem. Eur. J..

[98] Uenishi J., Ohmi M. (2005). Total synthesis of (−)-laulimalide: Pd-catalyzed stereospecific ring construction of the substituted 3,6-dihydro [2H] pyran units. Angew. Chem.-Int. Ed..

[99] Wender P.A., Hegde S.G., Hubbard R.D., Zhang L. (2002). Total synthesis of (−)-laulimalide. J. Am. Chem. Soc..

[100] Williams D.R., Mi L., Mullins R.J., Stites R.E. (2002). Synthesis of (−)-laulimalide: An agent for microtubule stabilization. Tetrahedron Lett..

[101] Bennett M.J., Chan G.K., Rattner J.B., Schriemer D.C. (2012). Low-dose laulimalide represents a novel molecular probe for investigating microtubule organization. Cell Cycle.

[102] Prota A.E., Bargsten K., Northcote P.T., Marsh M., Altmann K.H., Miller J.H., Fernando Díaz J., Steinmetz M.O. (2014). Structural basis of microtubule stabilization by laulimalide and peloruside A. Angew. Chem.-Int. Ed..

[103] West L.M., Northcote P.T., Battershill C.N. (2000). Peloruside A: A potent cytotoxic macrolide isolated from the New Zealand marine sponge *Mycale* sp.. J. Org. Chem..

[104] Hood K.A., West L.M., Rouwé B., Northcote P.T., Berridge M.V., Wakefield J., Miller J.H. (2002). Peloruside A, a novel antimitotic agent with paclitaxel-like microtubule-stabilizing activity. Cancer Res..

[105] Meyer C.J., Krauth M., Wick M.J., Shay J.W., Gellert G., De Brabander J.K., Northcote P.T., Miller J.H. (2015). Peloruside A inhibits growth of human lung and breast tumor xenografts in an athymic *nu/nu* mouse model. Mol. Cancer Ther..

[106] Gewirtz D.A., Holt S.E., Elmore L.W. (2008). Accelerated senescence: An emerging role in tumor cell response to chemotherapy and radiation. Biochem. Pharmacol..

[107] Chan A., Gilfillan C., Templeton N., Paterson I., Northcote P.T., Miller J.H. (2017). Induction of accelerated senescence by the microtubule-stabilizing agent peloruside A. Investig. New Drugs.

[108] Chan A., Singh A.J., Northcote P.T., Miller J.H. (2015). Inhibition of human vascular endothelial cell migration and capillary-like tube formation by the microtubule-stabilizing agent peloruside A. Investig. New Drugs.

[109] Das V., Miller J.H. (2012). Microtubule stabilization by peloruside A and paclitaxel rescues degenerating neurons from okadaic acid-induced tau phosphorylation. Eur. J. Neurosci..

[110] O’Sullivan D., Miller J.H., Northcote P.T., La Flamme A.C. (2013). Microtubule-stabilizing agents delay the onset of EAE through inhibition of migration. Immunol. Cell Biol..

[111] Crume K.P., O’Sullivan D., Miller J.H., Northcote P.T., La Flamme A.C. (2009). Delaying the onset of experimental autoimmune encephalomyelitis with the microtubule-stabilizing compounds, paclitaxel and Peloruside A. J. Leukoc. Biol..

[112] Singh A.J., Razzak M., Teesdale-Spittle P., Gaitanos T.N., Wilmes A., Paterson I., Goodman J.M., Miller J.H., Northcote P.T. (2011). Structure-activity studies of the pelorusides: New congeners and semi-synthetic analogues. Org. Biomol. Chem..

[113] Chany A.C., Legros F., Haroun H., Kundu U.K., Biletskyi B., Torlak S., Mathé-Allainmat M., Lebreton J., Macé A., Carboni B. (2019). Function-oriented synthesis toward peloruside A analogues. Org. Lett..

[114] Ghosh A.K., Xu X., Kim J.H., Xu C.X. (2008). Enantioselective total synthesis of peloruside A: A potent microtubule stabilizer. Org. Lett..

[115] Hoye T.R., Jeon J., Kopel L.C., Ryba T.D., Tennakoon M.A., Wang Y. (2010). Total synthesis of peloruside A through kinetic lactonization and relay ring-closing metathesis cyclization reactions. Angew. Chem. Int. Ed..

[116] Jin M., Taylor R.E. (2005). Total synthesis of (+)-peloruside A. Org. Lett..

[117] Liao X., Wu Y., De Brabander J.K. (2003). Total synthesis and absolute configuration of the novel microtubule-stabilizing agent peloruside A. Angew. Chem. Int. Ed..

[118] McGowan M.A., Stevenson C.P., Schiffler M.A., Jacobsen E.N. (2010). An enantioselective total synthesis of (+)-peloruside A. Angew. Chem. Int. Ed..

[119] Evans D.A., Welch D.S., Speed A.W.H., Moniz G.A., Reichelt A., Ho S. (2009). An aldol-based synthesis of (+)-peloruside A, a potent microtubule stabilizing agent. J. Am. Chem. Soc..

[120] Brackovic A., Harvey J.E. (2015). Synthetic, semisynthetic and natural analogues of peloruside A. Chem. Commun..

[121] Ranade A.R., Higgins L.A., Markowski T.W., Glaser N., Kashin D., Bai R., Hong K.H., Hamel E., Höfle G., Georg G.I. (2016). Characterizing the epothilone binding site on β-tubulin by photoaffinity labeling: Identification of β-tubulin peptides TARGSQQY and TSRGSQQY as targets of an epothilone photoprobe for polymerized tubulin. J. Med. Chem..

[122] Hardt I.H., Steinmetz H., Gerth K., Sasse F., Reichenbach H., Höfle G. (2001). New natural epothilones from *Sorangium cellulosum*, strains So ce90/B2 and So ce90/D13, Isolation, structure elucidation, and SAR studies. J. Nat. Prod..

[123] Gerth K., Steinmetz H., Höfle G., Reichenbach H. (2001). Studies on the biosynthesis of epothilones: The PKS and epothilone C/D monooxygenase. J. Antibiot. (Tokyo).

[124] Höfle G., Bedorf N., Steinmetz H., Schomburg D., Gerth K., Reichenbach H. (1996). Epothilone A and B—novel 16-membered macrolides with cytotoxic activity: Isolation, crystal structure, and conformation in solution. Angew. Chem. Int. Ed..

[125] Kowalski R.J., Giannakakou P., Hamel E. (1997). Activities of the microtubule-stabilizing agents epothilones A and B with purified tubulin and in cells resistant to paclitaxel (Taxol(R)). J. Biol. Chem..

[126] Rogalska A., Marczak A. (2018). Therapeutic potential of patupilone in epithelial ovarian cancer and future directions. Life Sci..

[127] Shen S., Kepp O., Martins I., Vitale I., Souquère S., Castedo M., Pierron G., Kroemer G. (2010). Defective autophagy associated with LC3 puncta in epothilone-resistant cancer cells. Cell Cycle.

[128] Rogalska A., Gajek A., Marczak A. (2019). Suppression of autophagy enhances preferential toxicity of epothilone A and epothilone B in ovarian cancer cells. Phytomedicine.

[129] Luu T., Kim K.P., Blanchard S., Anyang B., Hurria A., Yang L., Beumer J.H., Somlo G., Yen Y. (2018). Phase IB trial of ixabepilone and vorinostat in metastatic breast cancer. Breast Cancer Res. Treat..

[130] Rugo H.S., Roche H., Thomas E., Chung H.C., Lerzo G.L., Vasyutin I., Patel A., Vahdat L. (2018). Efficacy and safety of ixabepilone and capecitabine in patients with advanced triple-negative breast cancer: A pooled analysis from two large phase III, randomized clinical trials. Clin. Breast Cancer.

[131] Osborne C., Challagalla J.D., Eisenbeis C.F., Holmes F.A., Neubauer M.A., Koutrelakos N.W., Taboada C.A., Vukelja S.J., Wilks S.T., Allison M.A. (2018). Ixabepilone and carboplatin for hormone receptor positive/HER2-neu negative and triple negative metastatic breast cancer. Clin. Breast Cancer.

[132] Peethambaram P.P., Hartmann L.C., Jonker D.J., de Jonge M., Plummer E.R., Martin L., Konner J., Marshall J., Goss G.D., Teslenko V. (2015). A phase I pharmacokinetic and safety analysis of epothilone folate (BMS-753493), a folate receptor targeted chemotherapeutic agent in humans with advanced solid tumors. Investig. New Drugs.

[133] Gaugaz F.Z., Chicca A., Redondo-Horcajo M., Barasoain I., Fernando Díaz J., Altmann K.H. (2019). Synthesis, microtubule-binding affinity, and antiproliferative activity of new epothilone analogs and of an EGFR-targeted epothilone-peptide conjugate. Int. J. Mol. Sci..

[134] Brunden K.R., Zhang B., Carroll J., Yao Y., Potuzak J.S., Hogan A.M.L., Iba M., James M.J., Xie S.X., Ballatore C. (2010). Epothilone D improves microtubule density, axonal integrity, and cognition in a transgenic mouse model of tauopathy. J. Neurosci..

[135] Ruschel J., Hellal F., Flynn K.C., Dupraz S., Elliott D.A., Tedeschi A., Bates M., Sliwinski C., Brook G., Dobrindt K. (2015). Axonal regeneration. Systemic administration of epothilone B promotes axon regeneration after spinal cord injury. Science.

[136] Ye W., Liu T., Zhu M., Zhang W., Huang Z., Li S., Li H., Kong Y., Chen Y. (2019). An easy and efficient strategy for the enhancement of epothilone production mediated by TALE-TF and CRISPR/dcas9 systems in *Sorangium cellulosum*. Front. Bioeng. Biotechnol..

[137] Julien B., Shah S. (2002). Heterologous expression of epothilone biosynthetic genes in *Myxococcus xanthus*. Antimicrob. Agents Chemother..

[138] Tang L., Shah S., Chung L., Carney J., Katz L., Khosla C., Julien B. (2000). Cloning and heterologous expression of the epothilone gene cluster. Science.

[139] Mutka S.C., Carney J.R., Liu Y., Kennedy J. (2006). Heterologous production of epothilone C and D in *Escherichia coli*. Biochemistry.

[140] Wenzel S.C., Müller R. (2005). Recent developments towards the heterologous expression of complex bacterial natural product biosynthetic pathways. Curr. Opin. Biotechnol..

[141] Lau J., Frykman S., Regentin R., Ou S., Tsuruta H., Licari P. (2002). Optimizing the heterologous production of epothilone D in *Myxococcus xanthus*. Biotechnol. Bioeng..

[142] Ye W., Zhang W., Chen Y., Li H., Li S., Pan Q., Tan G., Liu T. (2016). A new approach for improving epothilone B yield in *Sorangium cellulosum* by the introduction of VGB epoF genes. J. Ind. Microbiol. Biotechnol..

[143] Martino E., Casamassima G., Castiglione S., Cellupica E., Pantalone S., Papagni F., Rui M., Siciliano A.M., Collina S. (2018). Vinca alkaloids and analogues as anti-cancer agents: Looking back, peering ahead. Bioorg. Med. Chem. Lett..

[144] Gigant B., Wang C., Ravelli R.B.G., Roussi F., Steinmetz M.O., Curmi P.A., Sobel A., Knossow M. (2005). Structural basis for the regulation of tubulin by vinblastine. Nature.

[145] Himes R.H. (1991). Interactions of the catharanthus (Vinca) alkaloids with tubulin and microtubules. Pharmacol. Ther..

[146] Lu K., Yap H.Y., Loo T.L. (1983). Clinical pharmacokinetics of vinblastine by continuous intravenous infusion. Cancer Res..

[147] Deyell R.J., Wu B., Rassekh S.R., Tu D., Samson Y., Fleming A., Bouffet E., Sun X., Powers J., Seymour L. (2019). Phase I study of vinblastine and temsirolimus in pediatric patients with recurrent or refractory solid tumors: Canadian cancer trials group study IND.218. Pediatr. Blood Cancer.

[148] Coiffier B., Lepage E., Brière J., Herbrecht R., Tilly H., Bouabdallah R., Morel P., Van Den Neste E., Salles G., Gaulard P. (2002). CHOP chemotherapy plus rituximab compared with CHOP alone in elderly patients with diffuse large-B-cell lymphoma. N. Engl. J. Med..

[149] Poeschel V., Held G., Ziepert M., Witzens-Harig M., Holte H., Thurner L., Borchmann P., Viardot A., Soekler M., Keller U. (2020). Four versus six cycles of CHOP chemotherapy in combination with six applications of rituximab in patients with aggressive B-cell lymphoma with favourable prognosis (FLYER): A randomised, phase 3, non-inferiority trial. Lancet.

[150] Meyer R.M., Gospodarowicz M.K., Connors J.M., Pearcey R.G., Wells W.A., Winter J.N., Horning S.J., Dar A.R., Shustik C., Stewart D.A. (2012). ABVD Alone versus Radiation-Based Therapy in Limited-Stage Hodgkin’s Lymphoma. N. Engl. J. Med..

[151] Waters E., Dingle B., Rodrigues G., Vincent M., Ash R., Dar R., Inculet R., Kocha W., Malthaner R., Sanatani M. (2010). Analysis of a novel protocol of combined induction chemotherapy and concurrent chemoradiation in unresected non-small-cell lung cancer: A ten-year experience with vinblastine, cisplatin, and radiation therapy. Clin. Lung Cancer.

[152] Zhigaltsev I.V., Maurer N., Akhong Q.F., Leone R., Leng E., Wang J., Semple S.C., Cullis P.R. (2005). Liposome-encapsulated vincristine, vinblastine and vinorelbine: A comparative study of drug loading and retention. J. Control. Release.

[153] Ling G., Zhang P., Zhang W., Sun J., Meng X., Qin Y., Deng Y., He Z. (2010). Development of novel self-assembled DS-PLGA hybrid nanoparticles for improving oral bioavailability of vincristine sulfate by P-gp inhibition. J. Control. Release.

[154] Leggans E.K., Duncan K.K., Barker T.J., Schleicher K.D., Boger D.L. (2013). A remarkable series of vinblastine analogues displaying enhanced activity and an unprecedented tubulin binding steric tolerance: C20’ urea derivatives. J. Med. Chem..

[155] Saba N., Seal A. (2018). Identification of a less toxic vinca alkaloid derivative for use as a chemotherapeutic agent, based on in silico structural insights and metabolic interactions with CYP3A4 and CYP3A5. J. Mol. Model.

[156] Va P., Campbell E.L., Robertson W.M., Boger D.L. (2010). Total synthesis and evaluation of a key series of C5-substituted vinblastine derivatives. J. Am. Chem. Soc..

[157] Bánóczi Z., Gorka-Kereskényi A., Reményi J., Orbán E., Hazai L., Tökési N., Oláh J., Ovádi J., Béni Z., Háda V. (2010). Synthesis and in vitro antitumor effect of vinblastine derivative-oligoarginine conjugates. Bioconjugate Chem..

[158] Manzo E., van Soest R., Matainaho L., Roberge M., Andersen R.J. (2003). Ceratamines A and B, antimitotic heterocyclic alkaloids isolated from the marine Sponge *Pseudoceratina* sp. collected in Papua New Guinea. Org. Lett..

[159] Tao L., Pan X., Ji M., Chen X., Liu Z. (2017). Efficient synthesis and cytotoxicity of novel microtubule-stabilizing agent ceratamine A analogues. Tetrahedron.

[160] Pan X., Tao L., Ji M., Chen X., Liu Z. (2018). Synthesis and cytotoxicity of novel imidazo[4,5-*d*]azepine compounds derived from marine natural product ceratamine A. Bioorg. Med. Chem. Lett..

[161] Nodwell M., Zimmerman C., Roberge M., Andersen R.J. (2010). Synthetic analogues of the microtubule-stabilizing sponge alkaloid ceratamine A are more active than the natural product. J. Med. Chem..

[162] Kowalski R.J., Giannakakou P., Gunasekera S.P., Longley R.E., Day B.W., Hamel E. (1997). The microtubule-stabilizing agent discodermolide competitively inhibits the binding of paclitaxel (Taxol) to tubulin polymers, enhances tubulin nucleation reactions more potently than paclitaxel, and inhibits the growth of paclitaxel-resistant cells. Mol. Pharmacol..

[163] Paterson I., Florence G.J., Carlomagno T. (2009). The chemical synthesis of discodermolide. Tubulin-Binding Agents: Synthetic, Structural and Mechanistic Insights.

[164] Pettit G., Kamano Y., Herald C.L., Tuinman A.A., Boettner F.E., Kizu H., Schmidt J.M., Baczynskyj L., Tomer K.B., Bontems R.J. (1987). The isolation and structure of a remarkable marine animal antineoplastic constituent: Dolastatin 10. J. Am. Chem. Soc..

[165] Bai R., Petit G.R., Hamel E. (1990). Dolastatin 10, a powerful cytostatic peptide derived from a marine animal: Inhibition of tubulin polymerization mediated through the vinca alkaloid binding domain. Biochem.Pharmacol..

[166] Garteiz D.A., Madden T., Beck D.E., Huie W.R., McManus K.T., Abbruzzese J.L., Chen W., Newman R.A. (1998). Quantitation of dolastatin-10 using HPLC/electrospray ionization mass spectrometry: Application in a phase I clinical trial. Cancer Chemother. Pharmacol..

[167] Pitot H.C., McElroy E.A., Reid J.M., Windebank A.J., Sloan J.A., Erlichman C., Bagniewski P.G., Walker D.L., Rubin J., Goldberg R.M. (1999). Phase I trial of dolastatin-10 (NSC 376128) in patients with advanced solid tumors. Clin. Cancer Res..

[168] von Mehren M., Balcerzak S.P., Kraft A.S., Edmonson J.H., Okuno S.H., Davey M., McLaughlin S., Beard M.T., Rogatko A. (2004). Phase II trial of dolastatin-10, a novel anti-tubulin agent, in metastatic soft tissue sarcomas. Sarcoma.

[169] Margolin K., Longmate J., Synold T.W., Gandara D.R., Weber J., Gonzalez R., Johansen M.J., Newman R., Baratta T., Doroshow J.H. (2001). Dolastatin-10 in metastatic melanoma: A phase II and pharmokinetic trial of the california cancer consortium. Investig. New Drugs.

[170] Hoffman M.A., Blessing J.A., Lentz S.S. (2003). A phase II trial of dolastatin-10 in recurrent platinum-sensitive ovarian carcinoma: A Gynecologic oncology group study. Gynecol. Oncol..

[171] Kindler H.L., Tothy P.K., Wolff R., McCormack R.A., Abbruzzese J.L., Mani S., Wade-Oliver K.T., Vokes E.E. (2005). Phase II trials of dolastatin-10 in advanced pancreaticobiliary cancers. Investig. New Drugs.

[172] Perez E.A., Hillman D.W., Fishkin P.A., Krook J.E., Tan W.W., Kuriakose P.A., Alberts S.R., Dakhil S.R. (2005). Phase II trial of dolastatin-10 in patients with advanced breast cancer. Investig. New Drugs.

[173] Maderna A., Doroski M., Subramanyam C., Porte A., Leverett C.A., Vetelino B.C., Chen Z., Risley H., Parris K., Pandit J. (2014). Discovery of cytotoxic dolastatin 10 analogues with N-terminal modifications. J. Med. Chem..

[174] Doronina S.O., Toki B.E., Torgov M.Y., Mendelsohn B.A., Cerveny C.G., Chace D.F., DeBlanc R.L., Gearing R.P., Bovee T.D., Siegall C.B. (2003). Development of potent monoclonal antibody auristatin conjugates for cancer therapy. Nat. Biotechnol..

[175] van de Donk N.W.C.J., Dhimolea E. (2012). Brentuximab vedotin. MAbs.

[176] Wagner S.M., Melchardt T., Egle A., Magnes T., Skrabs C., Staber P., Simonitsch-Klupp I., Panny M., Lehner B., Greil R. (2020). Treatment with brentuximab vedotin plus bendamustine in unselected patients with CD30-positive aggressive lymphomas. Eur. J. Haem..

[177] Fu J., Bian M., Jiang Q., Zhang C. (2007). Roles of aurora kinases in mitosis and tumorigenesis. Mol. Cancer Res..

[178] Magnaghi-Jaulin L., Eot-Houllier G., Gallaud E., Giet R. (2019). Aurora A protein kinase: To the centrosome and beyond. Biomolecules.

[179] Barr A.R., Gergely F. (2007). Aurora-A: The maker and breaker of spindle poles. J. Cell Sci..

[180] Krenn V., Musacchio A. (2015). The Aurora B kinase in chromosome bi-orientation and spindle checkpoint signaling. Front. Oncol..

[181] Yang K.T., Tang C.J.C., Tang T.K. (2015). Possible role of Aurora-C in meiosis. Front. Oncol..

[182] Liu Q., Kaneko S., Yang L., Feldman R.I., Nicosia S.V., Chen J., Cheng J.Q. (2004). Aurora-A abrogation of p53 DNA binding and transactivation activity by phosphorylation of serine 215. J. Biol. Chem..

[183] Borisa A.C., Bhatt H.G. (2017). A comprehensive review on Aurora kinase: Small molecule inhibitors and clinical trial studies. Eur. J. Med. Chem..

[184] Sells T.B., Chau R., Ecsedy J.A., Gershman R.E., Hoar K., Huck J., Janowick D.A., Kadambi V.J., LeRoy P.J., Stirling M. (2015). MLN8054 and alisertib (MLN8237): Discovery of selective oral aurora A inhibitors. ACS Med. Chem. Lett..

[185] Li J.P., Yang Y.X., Liu Q.L., Pan S.T., He Z.X., Zhang X., Yang T., Chen X.W., Wang D., Qiu J.X. (2015). The investigational Aurora kinase A inhibitor alisertib (MLN8237) induces cell cycle G(2)/M arrest, apoptosis, and autophagy via p38 MAPK and Akt/mTOR signaling pathways in human breast cancer cells. Drug Des. Devel. Ther..

[186] Fu Y., Zhang Y., Gao M., Quan L., Gui R., Liu J. (2016). Alisertib induces apoptosis and autophagy through targeting the AKT/mTOR/AMPK/p38 pathway in leukemic cells. Mol. Med. Rep..

[187] Ren B.J., Zhou Z.W., Zhu D.J., Ju Y.L., Wu J.H., Ouyang M.Z., Chen X.W., Zhou S.F. (2016). Alisertib induces cell cycle arrest, apoptosis, autophagy and suppresses EMT in HT29 and Caco-2 cells. Int. J. Mol. Sci..

[188] Liu Z., Wang F., Zhou Z.W., Xia H.C., Wang X.Y., Yang Y.X., He Z.X., Sun T., Zhou S.F. (2017). Alisertib induces G(2)/M arrest, apoptosis, and autophagy via PI3K/Akt/mTOR- and p38 MAPK-mediated pathways in human glioblastoma cells. Am. J. Transl. Res..

[189] Shang Y.Y., Yao M., Zhou Z.W., Cui J., Xia L., Hu R.Y., Yu Y.Y., Gao Q., Yang B., Liu Y.X. (2017). Alisertib promotes apoptosis and autophagy in melanoma through p38 MAPK-mediated aurora a signaling. Oncotarget.

[190] Zhu Q., Yu X., Zhou Z.W., Zhou C., Chen X.W., Zhou S.F. (2017). Inhibition of aurora A kinase by alisertib induces autophagy and cell cycle arrest and increases chemosensitivity in human hepatocellular carcinoma HepG2 cells. Curr. Cancer Drug Targets.

[191] Otto T., Horn S., Brockmann M., Eilers U., Schüttrumpf L., Popov N., Kenney A.M., Schulte J.H., Beijersbergen R., Christiansen H. (2009). Stabilization of N-Myc is a critical function of aurora A in human neuroblastoma. Cancer Cell.

[192] Niu H., Manfredi M., Ecsedy J.A. (2015). Scientific rationale supporting the clinical development strategy for the investigational Aurora A kinase inhibitor alisertib in cancer. Front. Oncol..

[193] Yang H., Liu J., Huang Y., Gao R., Tang B., Li S., He J., Li H. (2017). Domain-specific interactions between MLN8237 and human serum albumin estimated by STD and WaterLOGSY NMR, ITC, spectroscopic, and docking techniques. Sci. Rep..

[194] O’Connor O.A., Özcan M., Jacobsen E.D., Roncero J.M., Trotman J., Demeter J., Masszi T., Pereira J., Ramchandren R., Beaven A. (2019). Randomized phase III study of alisertib or investigator’s choice (selected single agent) in patients with relapsed or refractory peripheral T-cell lymphoma. J. Clin. Oncol..

[195] Shah H.A., Fischer J.H., Venepalli N.K., Danciu O.C., Christian S., Russell M.J., Liu L.C., Zacny J.P., Dudek A.Z. (2019). Phase I study of aurora A kinase inhibitor alisertib (MLN8237) in combination with selective VEGFR inhibitor pazopanib for therapy of advanced solid tumors. Am. J. Clin. Oncol..

[196] DuBois S.G., Mosse Y.P., Fox E., Kudgus R.A., Reid J.M., McGovern R., Groshen S., Bagatell R., Maris J.M., Twist C.J. (2018). Phase II trial of alisertib in combination with irinotecan and temozolomide for patients with relapsed or refractory neuroblastoma. Clin. Cancer Res..

[197] Currier M.A., Sprague L., Rizvi T.A., Nartker B., Chen C.Y., Wang P.Y., Hutzen B.J., Franczek M.R., Patel A.V., Chaney K.E. (2017). Aurora A kinase inhibition enhances oncolytic herpes virotherapy through cytotoxic synergy and innate cellular immune modulation. Oncotarget.

[198] Iankov I.D., Kurokawa C.B., D’Assoro A.B., Ingle J.N., Domingo-Musibay E., Allen C., Crosby C.M., Nair A.A., Liu M.C., Aderca I. (2015). Inhibition of the aurora A kinase augments the anti-tumor efficacy of oncolytic measles virotherapy. Cancer Gene Ther..

[199] Mortlock A.A., Foote K.M., Heron N.M., Jung F.H., Pasquet G., Lohmann J.J.M., Warin N., Renaud F., De Savi C., Roberts N.J. (2007). Discovery, synthesis, and in vivo activity of a new class of pyrazoloquinazolines as selective inhibitors of aurora B kinase. J. Med. Chem..

[200] Wilkinson R.W., Odedra R., Heaton S.P., Wedge S.R., Keen N.J., Crafter C., Foster J.R., Brady M.C., Bigley A., Brown E. (2007). AZD1152, a selective inhibitor of Aurora B kinase, inhibits human tumor xenograft growth by inducing apoptosis. Clin. Cancer Res..

[201] Zekri A., Mesbahi Y., Ghanizadeh-Vesali S., Alimoghaddam K., Ghavamzadeh A., Ghaffari S.H. (2017). Reactive oxygen species generation and increase in mitochondrial copy number: New insight into the potential mechanism of cytotoxicity induced by aurora kinase inhibitor, AZD1152-HQPA. Anticancer Drugs.

[202] Zekri A., Mesbahi Y., Boustanipour E., Sadr Z., Ghaffari S.H. (2018). The potential contribution of microRNAs in anti-cancer effects of aurora kinase inhibitor (AZD1152-HQPA). J. Mol. Neurosci..

[203] Ashton S., Song Y.S., Nolan J., Cadogan E., Murray J., Odedra R., Foster J., Hall P.A., Low S., Taylor P. (2016). Aurora kinase inhibitor nanoparticles target tumors with favorable therapeutic index in vivo. Sci. Transl. Med..

[204] Palmisiano N.D., Kasner M.T. (2015). Polo-like kinase and its inhibitors: Ready for the match to start?. Am. J. Hematol..

[205] Asghar U., Witkiewicz A.K., Turner N.C., Knudsen E.S. (2015). The history and future of targeting cyclin-dependent kinases in cancer therapy. Nat. Rev. Drug Discov..

[206] Colicino E.G., Hehnly H. (2018). Regulating a key mitotic regulator, polo-like kinase 1 (PLK1). Cytoskeleton.

[207] Yang X., Li H., Zhou Z., Wang W.H., Deng A., Andrisani O., Liu X. (2009). Plk1-mediated phosphorylation of topors regulates p53 stability. J. Biol. Chem..

[208] Goroshchuk O., Kolosenko I., Vidarsdottir L., Azimi A., Palm-Apergi C. (2019). Polo-like kinases and acute leukemia. Oncogene.

[209] López-Sánchez I., Sanz-García M., Lazo P.A. (2009). Plk3 interacts with and specifically phosphorylates VRK1 in Ser(342), a downstream target in a pathway that induces Golgi fragmentation. Mol. Cell. Biol..

[210] Habedanck R., Stierhof Y.D., Wilkinson C.J., Nigg E.A. (2005). The Polo kinase Plk4 functions in centriole duplication. Nat. Cell Biol..

[211] Ottmann O.G., Müller-Tidow C., Krämer A., Schlenk R.F., Lübbert M., Bug G., Krug U., Bochtler T., Voss F., Taube T. (2019). Phase I dose-escalation trial investigating volasertib as monotherapy or in combination with cytarabine in patients with relapsed/refractory acute myeloid leukaemia. Br. J. Haematol..

[212] Rudolph D., Steegmaier M., Hoffmann M., Grauert M., Baum A., Quant J., Haslinger C., Garin-Chesa P., Adolf G.R. (2009). BI 6727, A Polo-like kinase inhibitor with improved pharmacokinetic profile and broad antitumor activity. Clin. Cancer Res..

[213] Van den Bossche J., Deben C., de Pauw I., Lambrechts H., Hermans C., Deschoolmeester V., Jacobs J., Specenier P., Pauwels P., Vermorken J.B. (2019). In vitro study of the Polo-like kinase 1 inhibitor volasertib in non-small-cell lung cancer reveals a role for the tumor suppressor p53. Mol. Oncol..

[214] Solans B.P., Fleury A., Freiwald M., Fritsch H., Haug K., Trocóniz I.F. (2018). Population pharmacokinetics of volasertib administered in patients with acute myeloid leukaemia as a single agent or in combination with cytarabine. Clin. Pharm..

[215] Gumireddy K., Reddy M.V.R., Cosenza S.C., Boominathan R., Baker S.J., Papathi N., Jiang J., Holland J., Reddy E.P. (2005). 1ON01910, a non-ATP-competitive small molecule inhibitor of Plk1, is a potent anticancer agent. Cancer Cell.

[216] Steegmaier M., Hoffmann M., Baum A., Lénárt P., Petronczki M., Krssák M., Gürtler U., Garin-Chesa P., Lieb S., Quant J. (2007). BI 2536, a potent and selective inhibitor of Polo-like kinase 1, inhibits tumor growth in vivo. Curr. Biol..

[217] Ma W.W., Messersmith W.A., Dy G.K., Weekes C.D., Whitworth A., Ren C., Maniar M., Wilhelm F., Eckhardt S.G., Adjei A.A. (2012). Phase I study of rigosertib, an inhibitor of the phosphatidylinositol 3-kinase and Polo-like kinase 1 pathways, combined with gemcitabine in patients with solid tumors and pancreatic cancer. Clin. Cancer Res..

[218] Prasad A., Park I.W., Allen H., Zhang X., Reddy M.V.R., Boominathan R., Reddy E.P., Groopman J.E. (2009). Styryl sulfonyl compounds inhibit translation of cyclin D1 in mantle cell lymphoma cells. Oncogene.

[219] Castellano E., Downward J. (2011). RAS interaction with PI3K: More than just another effector pathway. Genes Cancer..

[220] Ritt D.A., Blanco M.A., Bindu L., Durrant D.E., Zhou M., Specht S.I., Stephen A.G., Holderfield M., Morrison D.K. (2016). Inhibition of Ras/Raf/MEK/ERK pathway signaling by a stress-induced phospho-regulatory circuit. Mol. Cell.

[221] Jost M., Chen Y., Gilbert L.A., Horlbeck M.A., Krenning L., Menchon G., Rai A., Cho M.Y., Stern J.J., Prota A.E. (2017). Combined CRISPRi/a-based chemical genetic screens reveal that rigosertib is a microtubule-destabilizing agent. Mol. Cell.

[222] Baker S.J., Cosenza S.C., Athuluri-Divakar S., Reddy M.V.R., Vasquez-Del Carpio R., Jain R., Aggarwal A.K., Reddy E.P. (2019). Mechanism of action of rigosertib does not involve tubulin binding. bioRxiv.

[223] Garcia-Manero G., Fenaux P., Al-Kali A., Baer M.R., Sekeres M.A., Roboz G.J., Gaidano G., Scott B.L., Greenberg P., Platzbecker U. (2016). Rigosertib versus best supportive care for patients with high-risk myelodysplastic syndromes after failure of hypomethylating drugs (ONTIME): A randomised, controlled, phase 3 trial. Lancet Oncol..

[224] Prasad A., Khudaynazar N., Tantravahi R.V., Gillum A.M., Hoffman B.S. (2016). ON 01910.Na (rigosertib) inhibits PI3K/Akt pathway and activates oxidative stress signals in head and neck cancer cell lines. Oncotarget.

[225] Anderson R.T., Keysar S.B., Bowles D.B., Glogowska M.J., Astling D.P., Morton J.P., Le P., Umpierrez A., Eagles-Soukup J., Gan G.N. (2013). The dual pathway inhibitor rigosertib is effective in direct-patient tumor xenografts of head and neck squamous cell carcinomas. Mol. Cancer Ther..

[226] Rice S., Lin A.W., Safer D., Hart C.L., Naber N., Carragher B.O., Cain S.M., Pechatnikova E., Wilson-Kubalek E.M., Whittaker M. (1999). A structural change in the kinesin motor protein that drives motility. Nature.

[227] Hepperla A.J., Willey P.T., Coombes C.E., Schuster B.M., Gerami-Nejad M., McClellan M., Mukherjee S., Fox J., Winey M., Odde D.J. (2014). Minus-end-directed kinesin-14 motors align antiparallel microtubules to control metaphase spindle length. Dev. Cell.

[228] Chen Y., Hancock W.O. (2015). Kinesin-5 is a microtubule polymerase. Nat. Commun..

[229] Trofimova D., Paydar M., Zara A., Talje L., Kwok B.H., Allingham J.S. (2018). Ternary complex of Kif2A-bound tandem tubulin heterodimers represents a kinesin-13-mediated microtubule depolymerization reaction intermediate. Nat. Commun..

[230] Rath O., Kozielski F. (2012). Kinesins and cancer. Nat. Rev. Cancer.

[231] Huszar D., Theoclitou M.E., Skolnik J., Herbst R. (2009). Kinesin motor proteins as targets for cancer therapy. Cancer Metastasis Rev..

[232] Mayer T.U., Kapoor T.M., Haggarty S.J., King R.W., Schreiber S.L., Mitchison T.J. (1999). Small molecule inhibitor of mitotic spindle bipolarity identified in a phenotype-based screen. Science.

[233] Abnous K., Barati B., Mehri S., Reza M., Farimani M.R.M., Alibolandi M., Mohammadpour F., Ghandadi M., Hadizadeh F. (2013). Synthesis and molecular modeling of six novel monastrol analogues: Evaluation of cytotoxicity and kinesin inhibitory activity against HeLa cell line. DARU.

[234] Kaan H.Y.K., Ulaganathan V., Rath O., Prokopcová H., Dallinger D., Kappe C.O., Kozielski F. (2010). Structural basis for inhibition of Eg5 by dihydropyrimidines: Stereoselectivity of antimitotic inhibitors enastron, dimethylenastron and fluorastrol. J. Med. Chem..

[235] De Oliveira F.S., De Oliveira P.M., Farias L.M., Brinkerhoff R.C., Sobrinho R.C.M.A., Treptow T.M., D’Oca C.R.M., Marinho M.A.G., Hort M.A., Horn A.P. (2018). Synthesis and antitumoral activity of novel analogues monastrol-fatty acids against glioma cells. Medchemcomm.

[236] Lee C.W., Bélanger K., Rao S.C., Petrella T.M., Tozer R.G., Wood L., Savage K.J., Eisenhauer E.A., Synold T.W., Wainman N. (2008). A phase II study of ispinesib (SB-715992) in patients with metastatic or recurrent malignant melanoma: A National cancer institute of Canada clinical trials group trial. Investig. New Drugs.

[237] Tang P.A., Siu L.L., Chen E.X., Hotte S.J., Chia S., Schwarz J.K., Pond G.R., Johnson C., Colevas A.D., Synold T.W. (2008). Phase II study of ispinesib in recurrent or metastatic squamous cell carcinoma of the head and neck. Investig. New Drugs.

[238] Beer T.M., Goldman B., Synold T.W., Ryan C.W., Vasist L.S., Van Veldhuizen P.J., Dakhil S.R., Lara P.N., Drelichman A., Hussain M.H.A. (2008). Southwest oncology group phase II study of ispinesib in androgen-independent prostate cancer previously treated with taxanes. Clin. Genitourin. Cancer.

[239] Kantarjian H.M., Padmanabhan S., Stock W., Tallman M.S., Curt G.A., Li J., Osmukhina A., Wu K., Huszar D., Borthukar G. (2012). Phase I/II multicenter study to assess the safety, tolerability, pharmacokinetics and pharmacodynamics of AZD4877 in patients with refractory acute myeloid leukemia. Investig. New Drugs.

[240] LoRusso P.M., Goncalves P.H., Casetta L., Carter J.A., Litwiler K., Roseberry D., Rush S., Schreiber J., Simmons H.M., Ptaszynski M. (2015). First-in-human phase 1 study of filanesib (ARRY-520), a kinesin spindle protein inhibitor, in patients with advanced solid tumors. Investig. New Drugs.

[241] Holen K.D., Belani C.P., Wilding G., Ramalingam S., Volkman J.L., Ramanathan R.K., Vasist L.S., Bowen C.J., Hodge J.P., Dar M.M. (2011). A first in human study of SB-743921, a kinesin spindle protein inhibitor, to determine pharmacokinetics, biologic effects and establish a recommended phase II dose. Cancer Chemother. Pharmacol..

[242] Wakui H., Yamamoto N., Kitazono S., Mizugaki H., Nakamichi S., Fujiwara Y., Nokihara H., Yamada Y., Suzuki K., Kanda H. (2014). A phase 1 and dose-finding study of LY2523355 (litronesib), an Eg5 inhibitor, in Japanese patients with advanced solid tumors. Cancer Chemother. Pharmacol..

[243] Hollebecque A., Deutsch E., Massard C., Gomez-Roca C., Bahleda R., Ribrag V., Bourgier C., Lazar V., Lacroix L., Gazzah A. (2013). A phase I, dose-escalation study of the Eg5-inhibitor EMD 534085 in patients with advanced solid tumors or lymphoma. Investig. New Drugs.

[244] Holen K., DiPaola R., Liu G., Tan A.R., Wilding G., Hsu K., Agrawal N., Chen C., Xue L., Rosenberg E. (2012). A phase I trial of MK-0731, a Kinesin Spindle Protein (KSP) inhibitor, in patients with solid tumors. Investig. New Drugs.

[245] Wood K.W., Lad L., Luo L., Qian X., Knight S.D., Nevins N., Brejc K., Sutton D., Gilmartin A.G., Chua P.R. (2010). Antitumor activity of an allosteric inhibitor of centromere-associated protein-E. Proc. Natl. Acad. Sci. USA.

[246] Chung V., Heath E.I., Schelman W.R., Johnson B.M., Kirby L.C., Lynch K.M., Botbyl J.D., Lampkin T.A., Holen K.D. (2012). First-time-in-human study of GSK923295, a novel antimitotic inhibitor of centromere-associated protein E (CENP-E), in patients with refractory cancer. Cancer Chemother. Pharmacol..

[247] Kung P.P., Martinez R., Zhu Z., Zager M., Blasina A., Rymer I., Hallin J., Xu M., Carroll C., Chionis J. (2014). Chemogenetic evaluation of the mitotic kinesin CENP-E reveals a critical role in triple-negative breast cancer. Mol. Cancer Ther..

[248] Ohashi A., Ohori M., Iwai K., Nambu T., Miyamoto M., Kawamoto T., Okaniwa M. (2015). A novel time-dependent CENP-E inhibitor with potent antitumor activity. PLoS ONE.

[249] Yamane M., Sawada J.I., Ogo N., Ohba M., Ando T., Asai A. (2019). Identification of benzo[d]pyrrolo[2,1-b]thiazole derivatives as CENP-E inhibitors. Biochem. Biophys. Res. Commun..

[250] Peterková L., Kmoníčková E., Ruml T., Rimpelová S. (2020). Sarco/endoplasmic reticulum calcium ATPase inhibitors: Beyond anticancer perspective. J. Med. Chem..

[251] Jurášek M., Rimpelová S., Kmoníčková E., Drašar P., Ruml T. (2014). Tailor-made fluorescent trilobolide to study its biological relevance. J. Med. Chem..

[252] Jurášek M., Černohorská M., Řehulka J., Spiwok V., Sulimenko T., Dráberová E., Darmostuk M., Gurská S., Frydrych I., Buriánová R. (2018). Estradiol dimer inhibits tubulin polymerization and microtubule dynamics. J. Steroid. Biochem. Mol. Biol..

